# Evaluation of the Automated and Early Detection of Seasonal Epidemic Onset and Burden Levels (AEDSEO) method for respiratory surveillance using data from 21 European countries

**DOI:** 10.2807/1560-7917.ES.2026.31.30.2500896

**Published:** 2026-07-30

**Authors:** Sofia Myrup Otero, Hanne-Dorthe Emborg, Kasper Schou Telkamp, Ida Rask Moustsen-Helms, Bolette Søborg, Lasse Engbo Christiansen

**Affiliations:** 1 Statens Serum Institut (SSI), Copenhagen, Denmark; 2 Department of Epidemiology Research, Statens Serum Institut (SSI), Copenhagen, Denmark; 3 Department of Infectious Disease Epidemiology and Prevention, Statens Serum Institut (SSI), Copenhagen, Denmark

**Keywords:** seasonal surveillance, respiratory infections, onset detection, intensity levels, aedseo

## Abstract

**BACKGROUND:**

Seasonal respiratory pathogens place recurrent pressure on health services, creating a need for timely detection of seasonal onset and assessment of within-season intensity.

**AIM:**

We aimed to evaluate the Automated and Early Detection of Seasonal Epidemic Onset and Burden Levels (AEDSEO) method, developed using Danish respiratory surveillance data, externally.

**METHODS:**

Using 63 surveillance series of influenza, respiratory syncytial virus (RSV), acute respiratory infection (ARI) and influenza-like illness (ILI) from 21 European countries, season onset detection was compared with the Moving Epidemic Method (MEM), while intensity level categorisation was compared with MEM, the World Health Organization Average Curve Method (WHO-ACM) and the Mean Standard Deviation method (MSD).

**RESULTS:**

The AEDSEO method signalled onset in 60 of 63 surveillance series, while MEM crossed its epidemic threshold in 55 of 63. Among 55 surveillance series in which both methods signalled onset, AEDSEO signalled earlier in 45. Median lead times were 6.5 weeks for influenza, 4.5 for RSV, 22.0 for ARI and 5.5 for ILI. Growth after the AEDSEO onset signal was usually sustained, particularly for influenza, RSV and ILI. The AEDSEO method provided more consistent within-season intensity categorisation across seasons than MEM, WHO-ACM and MSD.

**CONCLUSION:**

Across diverse European respiratory surveillance series AEDSEO supported earlier detection of seasonal onset than MEM and more consistent within-season intensity categorisation than MEM, WHO-ACM and MSD. Its current operational use in the Danish national respiratory surveillance system further supports its practical applicability for timely situational assessment, planning, and communication of respiratory pathogen activity.

**Key public health message**

**What did you want to address in this study?**

Early detection of respiratory season onset and assessment of activity throughout the season can support timely public health action. However, these tasks can be challenging across heterogeneous respiratory surveillance series and in settings with limited or disrupted historical data. We describe and externally evaluate AEDSEO, an operational method developed in Denmark for early detection of respiratory season onset and assessment of within-season intensity.

**What have we learnt from this study?**

Overall, the Danish AEDSEO, detected seasonal onset earlier than Moving Epidemic Method (MEM) and provided broader and more interpretable within-season intensity classification than MEM, WHO-Average Curve Method and Mean Standard Deviation method. Seasonal onset performance was strongest for influenza and RSV, whereas within-season intensity categorisation was informative across a broader range of surveillance series.

**What are the implications of your findings for public health?**

These findings suggest that AEDSEO can support earlier situational awareness and more consistent interpretation of seasonal respiratory activity than commonly used benchmark methods. This may help public health authorities communicate current activity and prepare for increasing circulation. Activity levels may be presented across countries, but each level indicates how current activity compares with the historical activity of that specific surveillance series.

## Introduction

During the autumn and winter seasons in temperate regions, multiple respiratory pathogens circulate, causing infections and hospital admissions leading to pressure on healthcare services. The timing of seasonal activity varies between pathogens and seasons and the associated burden differs by pathogen and age group [1-4]. Surveillance systems therefore need to detect sustained increases in pathogen circulation and hospital admissions early enough to support planning and communication in the health sector.

Current methods for assessing respiratory-season activity include the Moving Epidemic Method (MEM) [5], the World Health Organization Average Curve Method (WHO-ACM) [6-8] and the Mean Standard Deviation method (MSD) [9]. The Moving Epidemic Method and WHO-ACM derive intensity levels from peak observations in historical seasons. They are therefore useful for comparing seasonal peaks, but less suited to assessing burden throughout the season. In addition, they require at least five historical seasons, which limits their use when surveillance data are sparse or have changed over time. There is no automatic adaptation to atypical historical seasons, instead users must decide whether outlier seasons and/or individual data points should be excluded. The Mean Standard Deviation method partly addresses this by using only the previous season, but at the cost of ignoring longer-term historical variability. For onset detection, MEM derives thresholds from historical pre-epidemic periods. Under default settings, atypical seasons may remain influential for up to 10 years, making onset detection sensitive to historical outliers. This can complicate interpretation when seasonal patterns are atypical or change over time, for example after major disruptions in circulation patterns [10,11].

The Automated and Early Detection of Seasonal Epidemic Onset and Burden Levels (AEDSEO) method was developed using Danish respiratory surveillance data. Since the 2024/25 season it has been part of the integrated Danish national respiratory surveillance system for weekly monitoring of seasonal onset and within-season intensity of influenza, respiratory syncytial virus (RSV), severe acute respiratory syndrome coronavirus 2 (SARS-CoV-2) and *Mycoplasma pneumoniae*. The method combines disease-specific early detection of sustained growth with intensity classification, allowing interpretation before the seasonal peak is reached. In this study, we formally describe AEDSEO and externally evaluate its performance using 63 respiratory surveillance series from 21 European countries, benchmarking onset timing against MEM and intensity categorisation against MEM, WHO-ACM and MSD.

## Methods

### Automated and Early Detection of Seasonal Epidemic Onset and Burden Levels method development

The AEDSEO method was first developed for operational use in Denmark on count-based laboratory-confirmed infections for influenza and RSV and related admissions from seasons 2010/11 to 2023/24. In this study, that workflow was formalised into a prespecified and automated method implemented in the R software aedseo package version 1.1.0 [12], enabling reproducible application across diverse surveillance series and external evaluation against the current methods MEM, WHO-ACM and MSD. For the external evaluation, the same framework was applied to both count-based and incidence-based surveillance series. All data were gathered as weekly time series, and weeks were used as the time unit throughout. To capture the whole period of autumn and winter in temperate regions, a season is defined from week 21 in a year to week 20 in the subsequent year.

### Onset detection

The AEDSEO method identifies seasonal onset from short-term exponential growth in weekly counts. For each week, a rolling quasi-Poisson generalised linear model (GLM) with log link was fitted to counts [*Y_t_*] observed during the current and previous 4 weeks, corresponding to the default 5-week window [13]. Within each rolling window, the week index [*t*] was defined as, *t *=* *0, … 4, with *t *=* *4 corresponding to the current week:


*Y_t_ ~ quasi – Poisson(μ_t_), log(μ_t_) = β_0_ + β_1_t*

When population denominators were available, weekly counts were reconstructed from the reported incidence and the logarithm of the corresponding population denominator was included as an offset. The intercept [*β*_0_] represents the baseline level within the window and the time coefficient [*β*_1_] represents short-term growth, meaning that a positive time coefficient indicates increasing expected counts over time. Quasi-Poisson regression was used to account for overdispersion in weekly counts and was selected as the default model because results were similar when negative-binomial models were used. Estimated growth was considered positive when the 95% confidence interval (CI) for the time coefficient [*β*_1_] was above zero. The CI was obtained using the confint method for glm objects from the R stats software package [14].

### Disease-specific threshold

The AEDSEO method does not declare onset from growth alone as isolated positive slopes can occur at low counts because of random variation. Seasonal onset therefore required both positive growth and activity above a disease-specific threshold. A season was considered eligible if it contained an identifiable seasonal peak with at least one pre-peak sequence of positive growth and at least 3 significant weeks, allowing a gap of up to 1 week. For each pathogen or syndrome, up to the three most recent eligible seasons were examined. For each eligible season, the longest qualifying pre-peak sequence was selected as the candidate sequence. The corresponding rolling 5-week mean at the start of each sequence was determined. These values were then combined using exponentially decaying seasonal weights, with the greatest weight assigned to the most recent season, and the disease-specific threshold was defined as the 25th percentile of the weighted distribution. For count-based series, the disease-specific threshold was defined on the count scale and for incidence-based series, it was defined on the incidence scale. In prospective use, seasonal onset was declared the first week for which the rolling 5-week mean exceeded the disease-specific threshold and the lower 95% confidence bound for the time coefficient was greater than zero. This framework was implemented as default in the *estimate_disease_threshold* function in the R software aedseo package [12]. The function was applied for the external evaluation of the disease-specific threshold for all 63 surveillance series for the 2024/25 season; Supplement S1 presents a computational note describing how the function was used.

### Within-season intensity classification

To classify within-season intensity, the AEDSEO method used all available previous seasons indexed by i = 1, … N, with greater weight assigned to the most recent seasons. For each historical season [*i*], up to the three highest values above the disease-specific threshold were selected [*P_ij_*]. Seasons without values above the disease-specific threshold therefore did not contribute to the estimation of intensity breakpoints. Intensity breakpoints were estimated on the native reporting scale of each surveillance series. A weighted log-normal distribution with parameters *Θ *was then fitted to these selected peak values using exponentially decaying seasonal weights [*w_i_*] such that most recent seasons contributed more strongly, while still retaining longer-term historical information.


$$L(\theta )=-\sum_{i=1}^{N}\sum_{j=1}^{n_{i}}\mathrm{log f}(P_{ij}|\theta )\times w_{i},w_{i}=\alpha^{N-i},\alpha =0.8$$

The high breakpoint was defined as the 97.5th percentile of the fitted weighted log-normal distribution with estimated parameters and the very low breakpoint by the disease-specific threshold. The low and medium breakpoints were placed at equal distances on the log scale between the disease-specific threshold and the high breakpoint, yielding five intensity categories: very low, low, medium, high and very high.

For the external evaluation, AEDSEO onset and within-season intensity outputs were generated using the *combined_seasonal_output* function in the R software aedseo package [12], with the disease-specific thresholds estimated as described above. Code documentation on how aedseo was used for the external evaluation is available in Supplement S1.

### Parameter selection

Default parameter settings were initially informed by simulated seasonal time series and Danish surveillance data. For the present study, these settings were further assessed using targeted sensitivity analyses on European influenza and RSV surveillance series. These analyses examined how the rolling-window lengths (3–8 weeks) affected the estimated disease-specific threshold and the resulting onset timing, and how the number of peak values (1–8) selected from each historical season affected estimation of intensity breakpoints. The final defaults were a 5-week rolling window for growth estimation and disease-specific threshold derivation, and selection of three peak values per historical season for intensity modelling. A 5-week window reduced isolated false-positive onset detections while remaining responsive to sustained early increases. Selecting three peak values per season balanced sensitivity to unusually high single values against overly low intensity breakpoints when too many values were included. Candidate settings and corresponding results are reported in Supplement S2–S3.

The default decay factor on 0.8 [*α*] for the weights in the intensity level algorithm was chosen to balance responsiveness to recent seasons with stability across years and corresponds to an effective memory of approximately five seasons under exponential weighting [15].

### Benchmark methods

The AEDSEO method was benchmarked against three established methods: MEM [5], WHO-ACM [6-8] and MSD [9]. The Moving Epidemic Method provides both an epidemic threshold and seasonal intensity levels and was therefore used as the comparator for both onset detection and intensity categorisation. The World Health Organization Average Curve Method and MSD provide intensity levels only and were therefore used as comparators for intensity categorisation. The Moving Epidemic Method was implemented using the R software mem package [16] with otherwise standard settings. Following current respiratory surveillance practice, the 2020/21 season was excluded from the MEM historical window because circulation patterns during the COVID-19 pandemic were atypical. The World Health Organization Average Curve Method was applied using the geometric option for skewed peaks. When this yielded missing intensity breakpoints because log-transformation was not possible, the standard option was used instead. The Mean Standard Deviation method was implemented as described in the original method paper [9].

### Comparative evaluation of onset timing

Because no gold standard exists for the true onset of a seasonal epidemic wave, AEDSEO and MEM were compared by the timing of their onset signals. For surveillance series in which both methods signalled, the number of weeks between AEDSEO seasonal onset and the MEM epidemic threshold was calculated. As a comparative plausibility measure, growth in the intervening period was assessed to determine whether activity continued to increase after the earliest of the two signals. Sustained growth after the earliest signal was interpreted as support for earlier detection of seasonal increase. We calculated 95% CIs for binomial proportions using the Wilson method [17].

### External evaluation using European Respiratory Virus Surveillance Summary data

The European Respiratory Virus Surveillance Summary (ERVISS), a joint initiative of European Centre for Disease Prevention and Control (ECDC) and the WHO Regional Office for Europe, provides weekly country-level respiratory surveillance series collected through several surveillance systems [18]. Two types of surveillance-systems, count-based and incidence-based, were downloaded from ECDC’s GitHub [19] to externally evaluate AEDSEO:

(i) Sentinel syndromic data (incidence-based). Observations of influenza-like illness (ILI) and acute respiratory infection (ARI) that rely on syndromic case definitions. In most countries, ILI/ARI surveillance is conducted by sentinel networks of primary-care practices intended to be nationally representative, though coverage may vary. In some countries, surveillance is universal, covering the entire population [18].

(ii) Non-sentinel laboratory data (count-based). National diagnostic and reference laboratories report weekly totals of laboratory-confirmed detections of influenza virus and RSV irrespective of the patient’s clinical presentation or testing venue (hospital, outpatient clinic etc.) [18].

Countries were eligible for inclusion if they had at least five seasons of data, included both influenza and RSV series, had an average of at least 15 reporting weeks per season and contained data for the 2024/25 season. Influenza-like illness and ARI series were reported as incidence per 100,000 population, except for two countries that reported per 100 consultations. These countries were excluded because historical consultation denominators were not publicly available. Twenty-one countries met the criteria, contributing 63 surveillance series (21 influenza, 21 RSV, 8 ARI and 13 ILI). Countries were anonymised and are listed in random order in Supplement S4.

For onset detection, incidence-based ILI and ARI series were converted to weekly counts using country-specific population data from Eurostat [20], allowing the growth model to be fitted with a population offset. For disease-threshold estimation and within-season intensity classification, data were analysed on their native reporting scale.

## Results

**Figure 1:**
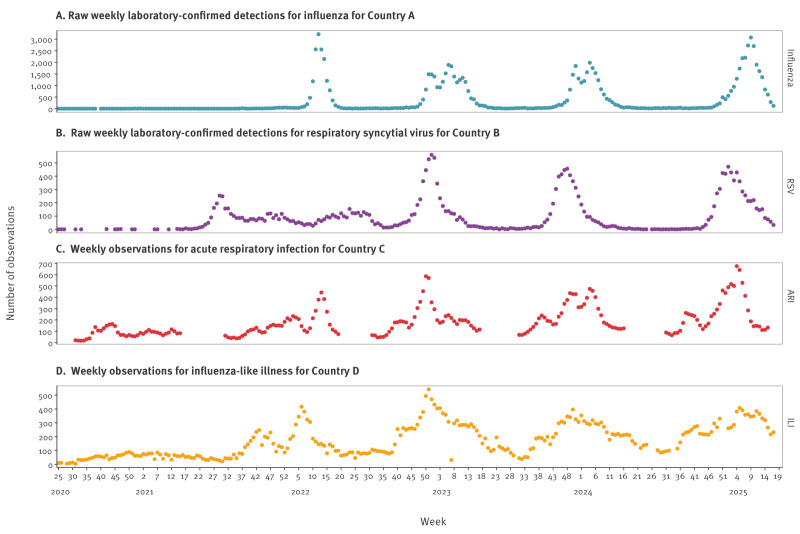
Representative surveillance series for raw weekly laboratory-confirmed detections for A) influenza and B) respiratory syncytial virus, and weekly observations of incidence per 100,000 population for C) acute respiratory infection and D) influenza-like illness, anonymised European countries A, B, C and D, seasons 2020/21 to 2024/25

**Figure 2:**
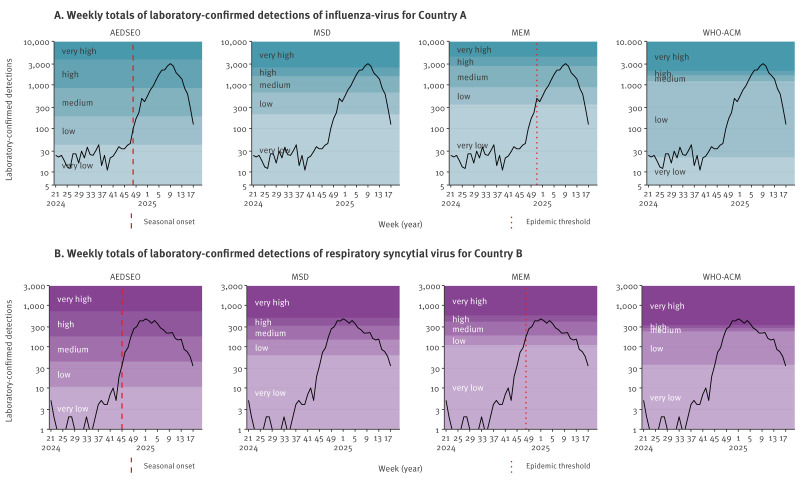
Comparing four different surveillance methods on non-sentinel laboratory data for weekly totals of laboratory-confirmed detections of A) influenza virus and B) respiratory syncytial virus, anonymised European countries A and B, season 2024/25

AEDSEO: Automated and Early Detection of Seasonal Epidemic Onset and Burden Levels; MEM: the Moving Epidemic Method; MSD: the Mean Standard Deviation method; WHO-ACM: the World Health Organization Average Curve Method.

**Figure 3:**
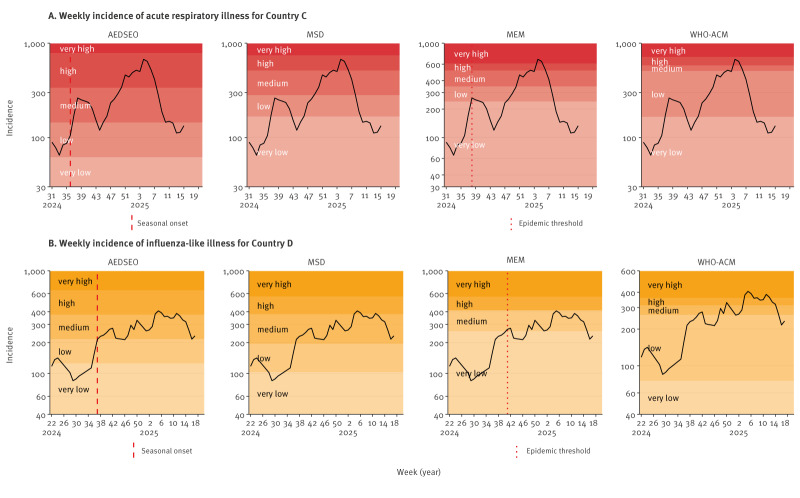
Comparing four different surveillance methods on sentinel syndromic data for weekly incidence per 100,000 population of A) acute respiratory infection and B) influenza-like illness, anonymised European countries C and D, season 2024/25

AEDSEO: Automated and Early Detection of Seasonal Epidemic Onset and Burden Levels; MEM: the Moving Epidemic Method; MSD: the Mean Standard Deviation method; WHO-ACM: the World Health Organization Average Curve Method.

Across the 63 surveillance series, influenza and RSV generally showed clearer winter seasonality than ARI and ILI, whereas ARI and ILI more often displayed broader or multiple peaks (Supplement S4). Notably, the 2020/21 season displays almost no detectable activity in influenza and RSV, likely due to the COVID-19 pandemic and associated non-pharmaceutical interventions [18,19]. Figures 1,2,3 and Table 1 show four representative surveillance series: non-sentinel influenza detections (Country A), non-sentinel RSV detections (Country B), sentinel ARI incidence (Country C) and sentinel ILI incidence (Country D).

**Table 1:** The highest peak values of influenza, respiratory syncytial virus, acute respiratory infection and influenza-like illness detected in seasons 2010/11 to 2023/24, and the peak values observed in season 2024/25, anonymised European countries A, B, C and D

Surveillance series	Historical highest peak season	Historical highest peak value	Peak value season 2024/25
Influenza (Country A)	2021/22	3,214.0	3,071.0
RSV (Country B)	2022/23	560.0	471.0
ARI^a^ (Country C)	2022/23	586.2	675.0
ILI^a^ (Country D)	2022/23	541.3	406.8

ARI: acute respiratory infection; ILI: influenza-like illness; RSV: respiratory syncytial virus.

^a^ Incidence per 100,000 population.

### Estimating the disease-specific threshold

For the four representative surveillance series shown in Figure 1, the estimated disease-specific threshold was 42.4 detections for influenza (Country A), 10.8 detections for RSV (Country B), 62.2 observations per 100,000 population for ARI (Country C), and 126.1 observations per 100,000 population for ILI (Country D). Disease-specific thresholds and MEM epidemic thresholds for all 63 surveillance series in season 2024/25 are provided in Supplement S5.

### Benchmark

Figures 2 and 3 show onset detection/epidemic-threshold timing and intensity categorisation for the four representative surveillance series in season 2024/25. Results for all 63 surveillance series are provided in Supplement S4.

For influenza and RSV, the main difference between methods was not only the peak category itself, but also the width of the intermediate intensity bands. For MEM and MSD, and partly for WHO-ACM, low, medium and high bands were often compressed because previous seasons had steep peaks. This makes within-season distinctions less informative as weekly activity can move rapidly across narrow bands or remain in a broad low-intensity band for a long period before changing category. In contrast the AEDSEO method yielded broader within-season categories for these representative surveillance series.

Influenza-like illness and ARI represent symptom-based syndromes caused by multiple pathogens; consequently, their activity can fluctuate through the surveillance year and may exhibit multiple peaks. However, Figure 1 shows that their highest peaks coincided with the periods of peak influenza and RSV activity for countries C and D, respectively. Outside the influenza and RSV peak periods, activity is likely driven by other respiratory pathogens with weaker or different seasonal patterns. For ILI (Country D; Figure 3), MEM did not yield a distinct low-intensity band because the epidemic threshold exceeded the low breakpoint.

Supplement S4 provides a corresponding comparison of intensity bands across methods and all 63 surveillance series. In the majority of series, AEDSEO yielded broader within-season categories than MEM, MSD and WHO-ACM, whereas the latter methods more often produced compressed intermediate bands or, in some cases, no distinct low-intensity band.

A seasonal onset was detected in 60 of 63 surveillance series (95%; 95% CI: 87–98) by AEDSEO, while MEM crossed its epidemic threshold in 55 of 63 (87.3%; 95% CI: 76.9–93.4). Among the 55 surveillance series in which both methods signalled, AEDSEO signalled earlier in 45 of 55 (82%; 95% CI: 70–90), MEM earlier in 7 of 55 (13%; 95% CI: 6–24) and both methods signalled in the same week in 3 of 55 (6%; 95% CI: 2–15). Five additional surveillance series were signalled only by AEDSEO, whereas three were signalled by neither method. Country-level estimations of seasonal onset and epidemic threshold timing by AEDSEO and MEM are available in Supplement S6.

Table 2 summarises the 45 surveillance series (Supplement S6; Supplementary Table S6-1) in season 2024/25 in which both methods signalled but AEDSEO signalled earlier. Median non-significant intervening weeks were low for influenza, RSV and ILI, but higher for ARI. The median proportion of intervening weeks with significant growth was 0.92 for influenza, 1.00 for RSV, 0.87 for ILI and 0.36 for ARI. For influenza and RSV, AEDSEO signalled a median of 6.5 (IQR: 3–12.25) and 4.5 (IQR: 4–12.25) weeks before MEM, respectively. The seven surveillance series (3 influenza, 3 RSV, 1 ILI) in which MEM signalled earlier than AEDSEO had a median of 1 intermediate week for influenza and RSV and 2 for ILI (Supplement S6; Supplementary Table S6-2). Among the 45 surveillance series in which AEDSEO signalled earlier than MEM, all had at least one intervening week with significant growth. Twenty-four had no intervening non-significant weeks. Among the remaining 21, eight had more significant than non-significant intervening weeks, two had equal numbers of significant and non-significant intervening weeks and 11 had more non-significant than significant intervening weeks.

**Table 2:** Lead time between the Automated and Early Detection of Seasonal Epidemic Onset and Burden Levels (AEDSEO) method seasonal onset and the Moving Epidemic Method (MEM) epidemic threshold for the 45 surveillance series in which AEDSEO signalled earlier than the MEM, 20 European countries, season 2024/25

Surveillance series	n	Median lead time (weeks) between AEDSEO and MEM		Number of intervening weeks without significant growth		Proportion of intervening weeks with significant growth	
		Median	IQR	Median	IQR	Median	IQR
Influenza	16	6.5	3.0–12.3	1.0	0.0–5.0	0.9	0.4–1.0
RSV	14	4.5	4.0–12.3	0.0	0.0–4.5	1.0	0.7–1.0
ARI	5	22.0	11.0–29.0	14.0	4.0–20.0	0.4	0.3–0.6
ILI	10	5.5	3.0–14.3	1.5	0.0–4.8	0.8	0.6–1.0

AEDSEO: Automated and Early Detection of Seasonal Epidemic Onset and Burden Levels; ARI: acute respiratory infection; ILI: influenza-like illness; IQR: interquartile range; MEM: the Moving Epidemic Method; RSV: respiratory syncytial virus.

## Discussion

Across 63 respiratory surveillance series from 21 European countries, AEDSEO generally signalled seasonal onset earlier than MEM and provided broader and more interpretable within-season intensity categories than MEM, WHO-ACM and MSD. Because AEDSEO requires both sustained positive growth and activity above the disease-specific threshold, seasonal onset is typically identified while activity is still in the low-to-medium range rather than at high burden. This is relevant in practice, as it may provide earlier situational awareness of upcoming circulation and time for preparation while the accompanying weekly intensity category communicates the level of activity throughout the season. In Denmark, AEDSEO outputs are used in routine respiratory surveillance to support weekly interpretation and communication of respiratory pathogen activity and pathogen-specific admissions to health authorities, healthcare professionals and the public. This is particularly useful when absolute detection counts differ substantially between pathogens, because the intensity categories place current observations in a pathogen-specific historical context rather than encouraging direct interpretation of raw counts alone.

Previous work on onset detection has evaluated short-term influenza alerting methods based on surveillance data, including time-series, regression and cumulative sum approaches [21], as well as exponentially weighted moving average charts [22] and Bayesian methods [23]. More recent work has also proposed expert consensus-based approaches for evaluating influenza epidemic-onset algorithms [24]. The AEDSEO method similarly uses recent growth information for onset detection but differs by combining a rolling growth model with a disease-specific threshold and within-season intensity classification in a framework intended for heterogeneous respiratory surveillance series. The main contribution of the present study is the formalisation and external evaluation of an operational surveillance workflow that supports both earlier onset detection and within-season interpretation across heterogeneous respiratory surveillance series.

The overall patterns across the 63 surveillance series were also informative. Influenza and RSV generally showed clearer winter seasonality than ARI and ILI, whereas ARI and ILI more often displayed broader or multiple peaks. Correspondingly, support for earlier AEDSEO signals was strongest for influenza and RSV and weaker for the more heterogeneous ARI series. This suggests that the standard AEDSEO onset detection is best suited to surveillance series with a clearer single seasonal rise, while within-season intensity classification may be the more informative output for syndromic series with less regular seasonality. For such multi-peak series, the AEDSEO framework also allows for repeated onset detection once activity has returned below a prespecified intensity level, while using the same intensity classification framework. However, this extension was not the focus of the present external evaluation and should be assessed in future work.

The benchmark methods partly address different surveillance questions. The Moving Epidemic Method provides an epidemic threshold and is therefore the most relevant comparator for onset timing, whereas MEM, WHO-ACM and MSD all provide retrospective intensity benchmarks. Our results suggest that the most practically important difference between methods was not only the final peak category, but also their ability to describe changes in intensity throughout the season. In several surveillance series, especially for influenza and RSV, MEM, MSD, and partly WHO-ACM, yielded compressed low, medium and high bands, reducing the interpretability of within-season changes before and after the seasonal peak. The AEDSEO method more often produced broader intermediate categories, which may support more gradual interpretation of rising, sustained and declining activity throughout the season rather than only classification of the seasonal peak. Targeted sensitivity analyses further supported the default 5-week rolling window as a practical balance between early responsiveness and robustness for onset detection, and the use of three peak values per season as a pragmatic basis for intensity classification.

This study has several limitations. First, no independent reference standard for the true onset of a seasonal epidemic wave was available. Onset timing was therefore evaluated comparatively against MEM and by the growth pattern observed between signals, which supports interpretation of earlier signals but does not provide formal alarm-system characteristics. Second, the analyses were based on retrospectively downloaded surveillance series, so the study should be interpreted as an external benchmarking study rather than as a formal validation. Third, the benchmark methods use different historical windows under their standard implementations. We retained these standard implementations because the aim was to compare AEDSEO with established respiratory surveillance methods as they are commonly applied in practice, but this means that timing and intensity comparisons are not fully harmonised across methods. Finally, each individual surveillance series contributes a limited number of seasons and epidemic events. The strength of the study therefore lies more in the heterogeneity of 63 respiratory surveillance series from 21 countries than in repeated validation within a single surveillance series.

Comparability between surveillance series across countries also remains limited. Influenza-like illness and ARI may be more comparable between countries than laboratory detections, but differences in case definitions, healthcare-seeking behaviour and surveillance coverage still affect interpretation. For non-sentinel influenza and RSV detections, international comparison is further complicated by variation in testing volume, geographic coverage and reporting practices across countries and seasons. These types of surveillance series are therefore best interpreted primarily within each surveillance series over time rather than as a direct tool for comparing absolute activity across countries.

Extreme seasons may still influence intensity estimates in all methods, particularly when testing practices change substantially. The AEDSEO method mitigates this by weighting recent seasons more heavily, but atypical seasons may still affect estimated breakpoints. Future applications could include a combination of respiratory pathogens and admission series (as used in Denmark) where sufficiently long and stable historical data are available. More broadly, AEDSEO may be useful in surveillance dashboards and routine weekly situational reporting where early onset detection and intensity assessment are both relevant.

## Conclusion

The AEDSEO method supported earlier detection of seasonal onset than the MEM and more consistent within-season intensity categorisation than the MEM, WHO-ACM and MSD. Together with its operational use in Denmark, these findings support its use for timely situational awareness, planning and communication of respiratory pathogen activity.

## Data Availability

All data used in this study are publicly available from the European Centre for Disease Prevention and Control (ECDC) GitHub repository: EU-ECDC/Respiratory_viruses_weekly_data.

## Supplementary Data

7433000 Supplementary Material
