# Supplementary material for: Evaluation of the Automated and Early Detection of Seasonal Epidemic Onset and Burden Levels (AEDSEO) method for respiratory surveillance using data from 21 European countries
Source: Euro Surveill. 2026 Jul 30;31(30):2500896. doi: 10.2807/1560-7917.ES.2026.31.30.2500896 (PMC13428196; doi:10.2807/1560-7917.ES.2026.31.30.2500896)
Supplement: Supplementary Material [file 25-00896_OTERO_Supplement.pdf]

# Supplementary material for manuscript: ***Evaluation of the Automated and Early Detection of Seasonal Epidemic Onset and Burden Levels (AEDSEO) method for respiratory surveillance using data from 21 European countries***

This supplementary material is hosted by *Eurosurveillance* as supporting information alongside the article "*Evaluation of the Automated and Early Detection of Seasonal Epidemic Onset and Burden Levels (AEDSEO) method for respiratory surveillance using data from 21 European countries*", on behalf of the authors, who remain responsible for the accuracy and appropriateness of the content. The same standards for ethics, copyright, attributions and permissions as for the article apply. Supplements are not edited by *Eurosurveillance* and the journal is not responsible for the maintenance of any links or email addresses provided therein.

## Table of contents

Supplement S1 .......... 3
Load the aedseo library .......... 3
Estimate the disease-specific threshold .......... 3
Influenza and RSV detections .......... 3
Sentinel (ILI and ARI) data .......... 4
Run the aedseo algorithm .......... 5
Influenza and RSV detections .......... 5
Sentinel (ILI and ARI) data .......... 6
Supplement S2 .......... 7
Targeted sensitivity analysis on the disease-specific threshold and sliding window size .......... 7
Supplement S3 .......... 10
Targeted sensitivity analysis on selection of number of peak observations for intensity level estimates . 10
Supplement S4 .......... 11
Countries included .......... 11
Practical interpretation of intensity levels .......... 11
All onset and intensity plots for countries A-U, season 2024/25 .......... 11
Country A .......... 12
Country B .......... 13
Country C .......... 14
Country D .......... 15
Country E .......... 16
Country F .......... 17
Country G .......... 18
Country H .......... 19

Country J ........ 21
Country K ........ 22
Country L ........ 23
Country M ........ 24
Country O ........ 26
Country P ........ 27
Country Q ........ 28
Country R ........ 29
Country S ........ 30
Country T ........ 31
Country U ........ 32
Supplement S5 ........ 33
Disease-specific thresholds (AEDSEO) and epidemic thresholds (MEM) for countries A-U, season 2024/25 ........ 33
Supplement S6 ........ 35
Weekly timing of Seasonal onset (AEDSEO) versus epidemic threshold (MEM) ........ 35

# Supplement S1

*A computational note about AEDSEO default settings using the aedseo package for the AEDSEO article analysis is supplied in the following.*

## Load the aedseo library

```
library(aedseo)
```

## Estimate the disease-specific threshold

### Influenza and RSV detections

```
non_sentinel_threshold_df <- all_non_sentinel_data |>
  dplyr::group_by(countryname, DiseaseCode) |>
  dplyr::group_map(~ {
    country <- .y$countryname
    disease <- .y$DiseaseCode
    print(paste("Country:", country, "Disease:", disease))

    # Setup the time series data
    tsd <- aedseo::to_time_series(
      cases = .x$observation,
      time = .x$reference_time,
      time_interval = "weeks"
    )

    # Estimate the disease-specific threshold
    thresholds <- aedseo::estimate_disease_threshold(
      tsd = tsd,
      season_start = 21,
      season_end = 20,
      skip_current_season = TRUE,
      min_significant_time = 3,
      max_gap_time = 1,
      use_prev_seasons_num = 3,
      pick_significant_sequence = "longest",
      season_importance_decay = 0.8,
      conf_levels = 0.25
    )

    stats::set_names(list(thresholds), paste0(country, "_", disease))
  })
non_sentinel_threshold_df <- unlist(non_sentinel_threshold_df, recursive = FALS
E)

non_sentinel_thresholds <- purrr::imap_dfr(non_sentinel_threshold_df, function(
x, nm) {
  parts <- stringr::str_split_fixed(nm, "_", 2)
  country <- parts[,1]
  disease <- parts[,2]
  val <- x$disease_threshold
```

```
  tibble::tibble(country = country, disease = disease, value = val)
})
```

### Sentinel (ILI and ARI) data

```
sentinel_threshold_df <- all_sentinel_data |>
  dplyr::group_by(countryname, DiseaseCode) |>
  dplyr::group_map(~ {
    country <- .y$countryname
    disease <- .y$DiseaseCode
    print(paste("Country:", country, "Disease:", disease))

    # Setup the time series data
    tsd <- aedseo::to_time_series(
      incidence = .x$observation,
      population = .x$population,
      time = .x$reference_time,
      incidence_denominator = 100000,
      time_interval = "weeks"
    )

    # Estimate the disease-specific threshold
    thresholds <- aedseo::estimate_disease_threshold(
      tsd = tsd,
      season_start = 21,
      season_end = 20,
      skip_current_season = TRUE,
      min_significant_time = 3,
      max_gap_time = 1,
      use_prev_seasons_num = 3,
      pick_significant_sequence = "longest",
      season_importance_decay = 0.8,
      conf_levels = 0.25
    )

    stats::set_names(list(thresholds), paste0(country, "_", disease))
  })
sentinel_threshold_df <- unlist(sentinel_threshold_df, recursive = FALSE)

sentinel_thresholds <- purrr::imap_dfr(sentinel_threshold_df, function(x, nm) {
  parts <- stringr::str_split_fixed(nm, "_", 2)
  country <- parts[,1]
  disease <- parts[,2]
  val <- x$disease_threshold

  tibble::tibble(country = country, disease = disease, value = val)
})
```

## Run the aedseo algorithm

### Influenza and RSV detections

```
aedseo_df_non_sentinel <- purrr::pmap(non_sentinel_thresholds, function(disease
, country, value) {
  print(paste(disease, country, value))
  cur_data <- all_non_sentinel_data |> filter(DiseaseCode == disease, countryna
me == country)

  # Setup the time series data
  tsd <- aedseo::to_time_series(
    cases = cur_data$observation,
    time = cur_data$reference_time,
    time_interval = "week"
  )

  # Run the aedseo algorithm
  aedseo_algo <- aedseo::combined_seasonal_output(
    tsd = tsd,
    season_start = 21,
    season_end = 20,
    disease_threshold = value,
    only_current_season = TRUE,
    # Seasonal onset default parameters
    family = "quasipoisson",
    k = 5,
    level = 0.95,
    na_fraction_allowed = 0.4,
    # Intensity levels default parameters
    family_quant = "lnorm",
    method = "intensity_levels",
    n_peak = 3,
    conf_levels = 0.975,
    decay_factor = 0.8,
    optim_method = "Nelder-Mead",
    lower_optim = -Inf,
    upper_optim = Inf,
    multiple_waves = FALSE
  )

  stats::set_names(list(aedseo_algo), paste0(disease, "_", country))
})
aedseo_df_non_sentinel <- unlist(aedseo_df_non_sentinel, recursive = FALSE)
```

### Sentinel (ILI and ARI) data

```
aedseo_df_sentinel <- purrr::pmap(sentinel_thresholds, function(disease, countr
y, value) {
  print(paste(disease, country, value))
  cur_data <- all_sentinel_data |> filter(DiseaseCode == disease, countryname =
= country)

  # Setup the time series data
  tsd <- aedseo::to_time_series(
    incidence = cur_data$observation,
    population = cur_data$population,
    time = cur_data$reference_time,
    time_interval = "week"
  )

  # Run the aedseo algorithm
  aedseo_algo <- aedseo::combined_seasonal_output(
    tsd = tsd,
    season_start = 21,
    season_end = 20,
    disease_threshold = value,
    only_current_season = TRUE,
    # Seasonal onset default parameters
    family = "quasipoisson",
    k = 5,
    level = 0.95,
    na_fraction_allowed = 0.4,
    # Intensity levels default parameters
    family_quant = "lnorm",
    method = "intensity_levels",
    n_peak = 3,
    conf_levels = 0.975,
    decay_factor = 0.8,
    optim_method = "Nelder-Mead",
    lower_optim = -Inf,
    upper_optim = Inf,
    multiple_waves = FALSE
  )

  stats::set_names(list(aedseo_algo), paste0(disease, "_", country))
})
aedseo_df_sentinel <- unlist(aedseo_df_sentinel, recursive = FALSE)
```

# Supplement S2

## Targeted sensitivity analysis on the disease-specific threshold and sliding window size

It is important to be able to detect positive growth in weekly observations to be able to alert that the circulation of the pathogen has started. To examine the effect of the rolling window size used in AEDSEO onset detection, we performed a targeted sensitivity analysis on influenza and RSV surveillance series from the countries included in the external evaluation. It was not done for ARI and ILI, as these are not expected to show a seasonal pattern. The *estimate_disease_threshold* function from the aedseo R package was applied with default settings while varying the rolling window from 3 to 8 weeks. We then assessed how the resulting window-specific thresholds influenced onset timing relative to MEM's epidemic threshold. The purpose was to evaluate how the choice of rolling window size affected both the derived threshold and the behaviour of the onset algorithm. These analyses supported the default 5-week window as a balance between responsiveness to sustained early increases and robustness to isolated false-positive signals.

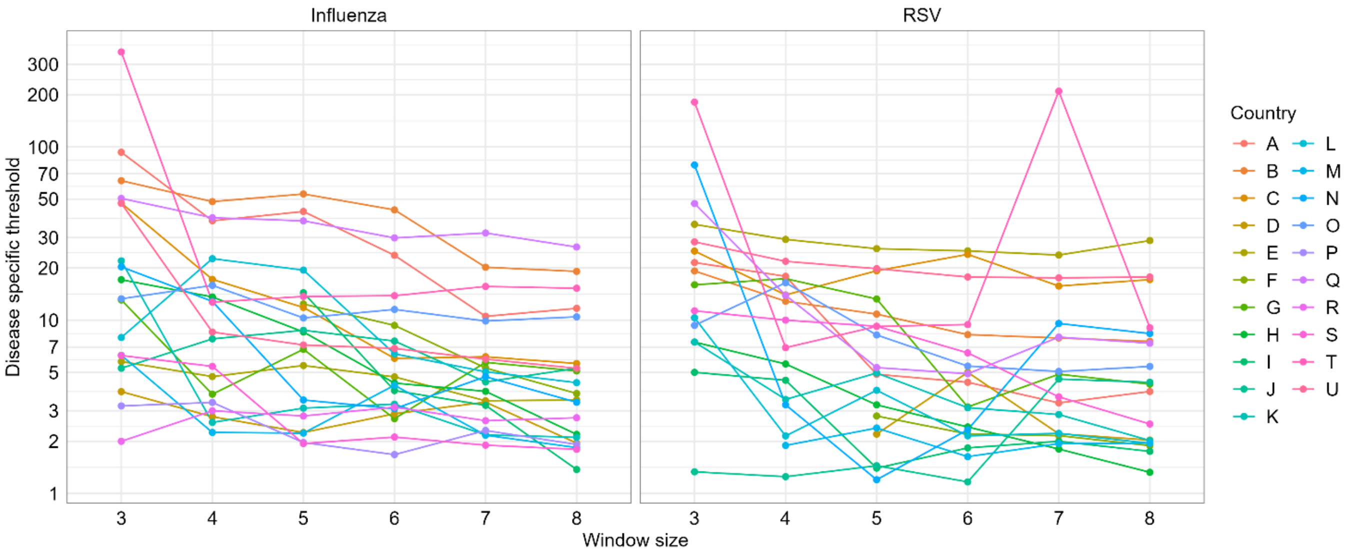


Figure S2. The figures show the disease-specific threshold estimated by the *estimate_disease_threshold* function in the aedseo R package as a function of window sizes (3-8) for influenza and RSV series from the 21 selected countries.

As AEDSEO uses 5 as the window size, 5 is selected as the baseline window size for comparison of the other window sizes (Figure S2). When increasing the window size from baseline, the disease-specific threshold decreases and contrary it increases if decreasing the window size (Table S2-1). If the disease-specific threshold is too high the seasonal onset might be called too late in the season, whereas if it is too low, we might have a too early false alarm. Accordingly, the natural next step was to investigate how the combination of window size and disease-specific threshold actually influence the seasonal onset.

| window | disease | threshold value shift median and IQR | seasonal onset shift median and IQR |
|---|---|---|---|
| 3 | Influenza | 6.3 (1.43 - 17.88) | 0.5 (0 - 2) |
| 4 | Influenza | 0.52 (-0.84 - 3.33) | 0 (0 - 1) |
| 5 | Influenza | 0 (0-0) | 0 (0 - 0) |
| 6 | Influenza | -0.76 (-5.82 - 0.17) | 0 (-1 - 1) |
| 7 | Influenza | -1.23 (-5.69 - (-0.06)) | 0 (-3 - 0.5) |
| 8 | Influenza | -1.92 (-8.62 - (-0.14)) | 0 (-3.5 - 0.25) |
| 3 | RSV | 6.09 (2.69 - 11.57) | 0.5 (0 - 3.25) |
| 4 | RSV | 2.03 (-0.49 - 3.4) | 0 (-0.75 - 0) |
| 5 | RSV | 0 (0 - 0) | 0 (0 - 0) |
| 6 | RSV | -0.73 (-1.95 - (-0.02)) | 0 (-1 - 0) |
| 7 | RSV | -1.54 (-2.62 - 0.31) | 0 (-0.25 - 1.75) |
| 8 | RSV | -1 (-2.47 - 0.1) | 0 (-1 - 2) |

*Table S2-1. The table includes information of the median and IQR shift in the disease-specific threshold and seasonal onset when increasing/decreasing the window size from baseline (window size = 5) grouped by influenza and RSV from the 21 selected countries.*

The *seasonal_onset* function from the aedseo R package was used with default parameters and varying the window size with its corresponding disease-specific threshold (Figure S2). Table S2-1 shows that the seasonal onset is in general called later when using a smaller window size than baseline, and in contrary it is called earlier when using a bigger window size, which coincides with the disease-specific threshold being very low when increasing window size (Figure S2).

| window | disease | Median (IQR) number of weeks between onset from AEDSEO to MEM | Median (IQR) number of significant weeks between onset from AEDSEO to MEM | Median (IQR) number of non-significant weeks between onset from AEDSEO to MEM |
|---|---|---|---|---|
| 3 | Influenza | 2 (1 - 6) | 2 (1.75 - 5) | 0 (0 - 3) |
| 4 | Influenza | 5 (3 - 10) | 3 (3 - 6) | 0 (0 - 3) |
| 5 | Influenza | 6 (2 - 11) | 4 (2 - 6.5) | 0 (0 - 3.5) |
| 6 | Influenza | 7 (3.5 - 11) | 5 (3.25 - 6.75) | 2.5 (0 - 3.75) |
| 7 | Influenza | 7 (4 - 12.5) | 5.5 (3.25 - 7) | 3 (0 - 7) |
| 8 | Influenza | 8 (6 - 12.5) | 5 (2.5 - 7.5) | 3 (1 - 7) |
| 3 | RSV | 2 (0 - 4) | 3 (2 - 3.75) | 0 (0 - 0) |
| 4 | RSV | 4 (2.75 - 7.5) | 4 (3 - 7.25) | 0 (0 - 0) |
| 5 | RSV | 4 (2 - 10) | 4 (3 - 7.25) | 0 (0 - 1.5) |
| 6 | RSV | 4 (3 - 10) | 4.5 (3.75 - 7.25) | 0 (0 - 2.25) |
| 7 | RSV | 4 (2 - 6) | 4 (3 - 6) | 0 (0 - 0) |
| 8 | RSV | 4 (2 - 7) | 4 (3.5 - 7) | 0 (0 - 0) |

Table S2-2. Includes information (median and IQR) of the number of weeks between seasonal onset detection by AEDSEO and MEM reaching the epidemic threshold. Same comparisons are made for window sizes 3-8 and estimations from the 21 countries are grouped by influenza and RSV.

A targeted sensitivity analysis was conducted for influenza and RSV across the 21 countries, by counting the number of weeks between when AEDSEO detects seasonal onset to when MEM reaches the epidemic

threshold and vice versa, while varying the AEDSEO window size between 3-8 weeks (Table S2-2). Across all window sizes, the median onset detected by AEDSEO occurred earlier than detected by MEM. The results show that increasing the window size generally leads to a larger time gap between the two methods.

For RSV, the number of significant weeks increases up to a window size of six weeks and then slightly declines, suggesting that very long windows begin to dilute early growth signals. For influenza, both the number of significant weeks and the number of non-significant weeks increase steadily with larger windows, implying that overly long windows may increase the risk of false detection. Consequently, selecting a window that is neither too short nor too long provides a better balance between early responsiveness and robustness to random variation.

Based on literature very short windows (≤ 2 weeks) detect significant growth earlier but are dominated by sampling variability, often masking the underlying temporal pattern. Very long windows (≥ 6 weeks) suppress that noise, yet they might postpone detection until the season is already well under way. This bias-variance trade-off is well documented [13], and our own sensitivity analysis shows the same (Table S2-2). A simulation study using the *generate_seasonal_data* function from the aedseo R package (data not shown) confirmed these findings, with bootstrapping of 200 × 15 simulated seasons yielding consistent results. Considering these findings and given that observations are reported weekly, we adopted a 5-week window (approx. 35 days), which is long enough to smooth random fluctuations and reveal sustained trends, while remaining sensitive to early emerging changes.

# Supplement S3

## Targeted sensitivity analysis on selection of number of peak observations for intensity level estimates

To examine the effect of the number of peak values selected from each historical season, we performed a targeted sensitivity analysis on influenza and RSV surveillance series from the countries included in the external evaluation. It was not done for ARI and ILI, as these are not expected to show a seasonal pattern The number of selected peak values was varied from 1 to 8, and for each setting we estimated the 97.5th percentile used to define the high breakpoint. The purpose was to assess how this choice affected the high intensity breakpoint. Across surveillance series, selecting more peak values lowered the estimated high breakpoint, whereas selecting very few values increased sensitivity to unusually high single observations. These analyses supported the default of three peak values per historical season as a pragmatic balance.

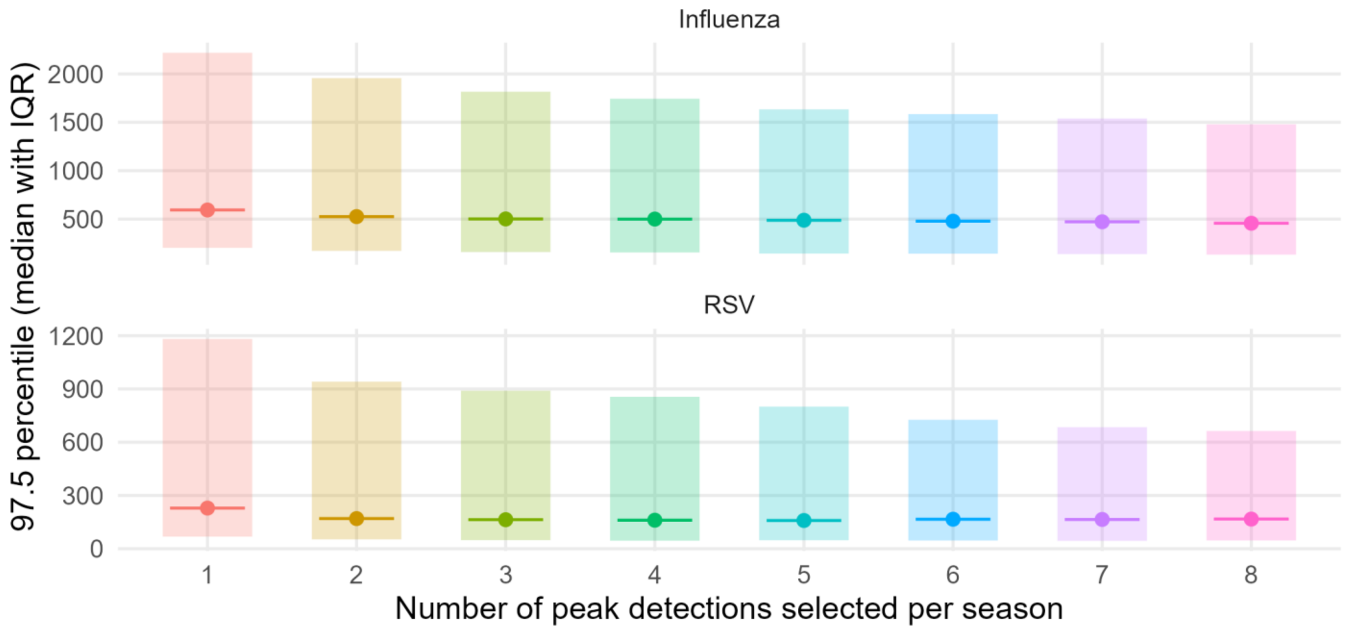


Figure S3. The figure shows the distribution of estimated 97.5th percentiles (median and IQR across all seasons for influenza and RSV and 21 European countries) versus the number of peak values (1-8) used in the high breakpoint estimation by AEDSEO.

When varying the number of peak values used to estimate the 97.5th percentile using AEDSEO, we observe a consistent pattern across both influenza and RSV (Figure S3), where increment in number of peak values lead to lower percentiles, hence a lower high breakpoint. Including more peaks therefore lowers the estimated 97.5th percentile (risking underestimation of true high-intensity seasons), while too few peaks lead to bias from e.g., extreme years or more testing.

Despite large heterogeneity across 21 European countries, with substantial heterogeneity in detection definitions, reporting systems, and data quality across seasons and diseases, the same systematic pattern was observed (Figure S3). This trend was confirmed using the *generate_seasonal_data* function from the aedseo R package (data not shown), with bootstrapping of 200 × 15 simulated seasons. This indicates that the underlying behaviour of the method is robust even under real-world surveillance variability. To balance this bias-variance trade-off, three peak detections from each season are used in the AEDSEO method.

# Supplement S4

## Countries included

Belgium, Bulgaria, Croatia, Czechia, Denmark, Estonia, France, Germany, Hungary, Iceland, Ireland, Latvia, Luxembourg, Malta, Netherlands, Poland, Portugal, Slovakia, Slovenia, Spain and Sweden. Countries are randomly anonymised as A–U throughout the manuscript and supplementary material at ECDC's request.

## Practical interpretation of intensity levels

- **very low** reflects inter-epidemic/background activity
- **low** indicates activity above baseline but still modest
- **medium** indicates typical moderate seasonal activity
- **high** indicates activity approaching the upper historical range
- **very high** indicates activity above the 97.5th percentile of the weighted historical peak distribution

## All onset and intensity plots for countries A-U, season 2024/25

Methods compared:

- Automated and Early Detection of Seasonal Epidemic Onset and Burden Levels (AEDSEO)
- Mean Standard Deviation method (MSD)
- Moving Epidemic Method (MEM)
- World Health Organization Average Curve Method (WHO-ACM)

Following plots compare the listed methods using weekly time series data from the eligible surveillance series and seasons for each European country (A-U). Influenza and RSV are reported as laboratory-confirmed detections. ARI and ILI are reported as observations per 100,000 population

# Country A

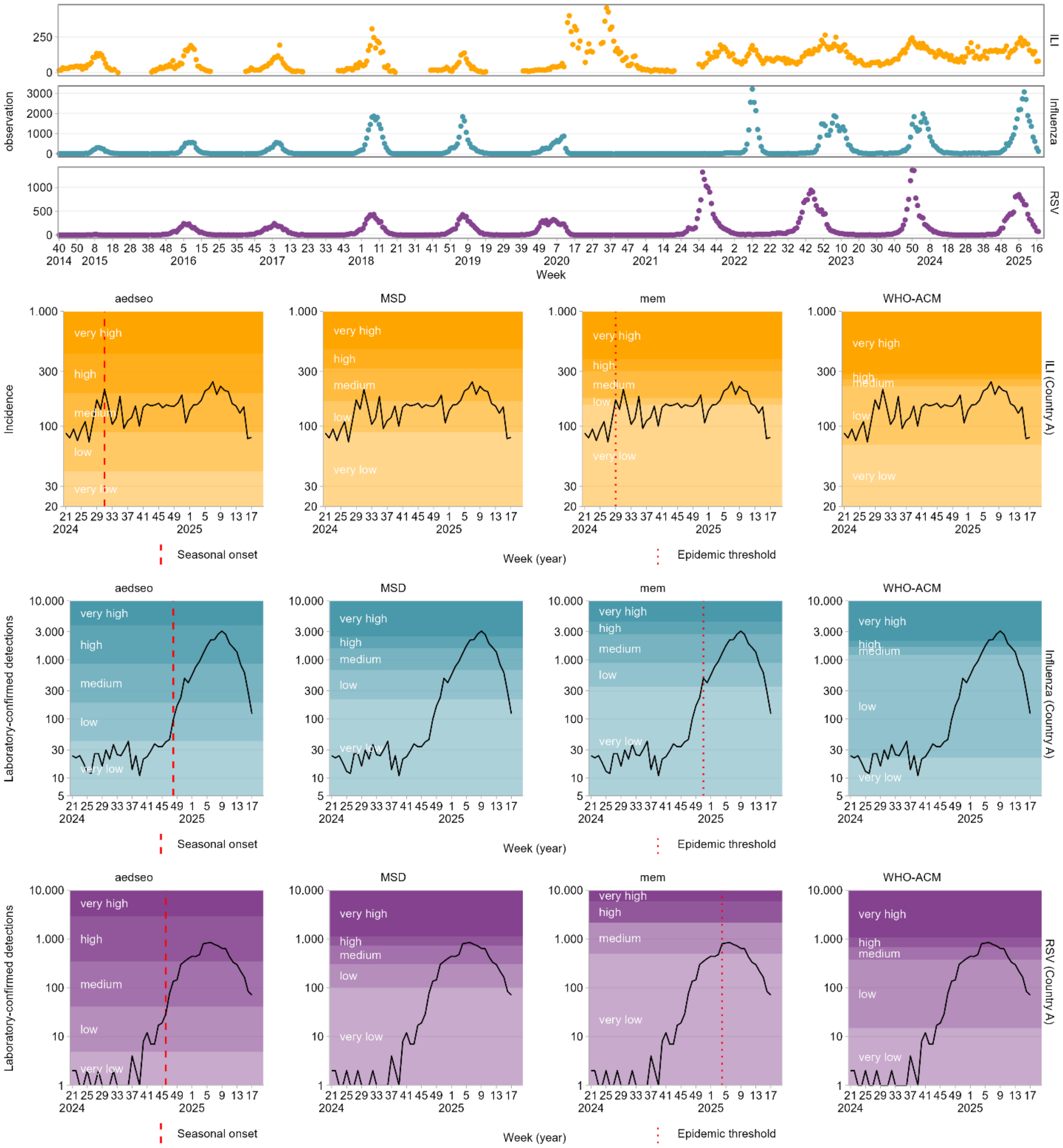

ILI
Influenza
RSV
observation
Week
aedseo
MSD
mem
WHO-ACM
Incidence
Laboratory-confirmed detections
ILI (Country A)
Influenza (Country A)
RSV (Country A)
very high
high
medium
low
very low
Week (year)
Seasonal onset
Epidemic threshold

## Country B

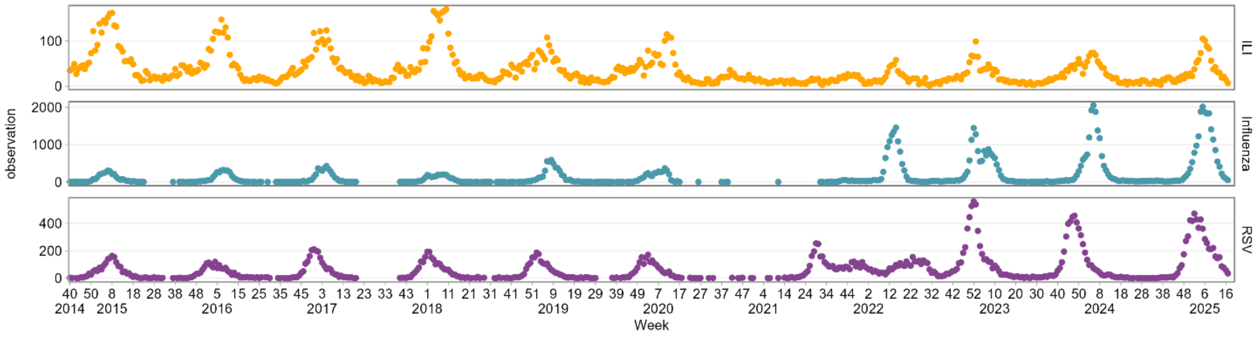

observation
ILI
Influenza
RSV
Week


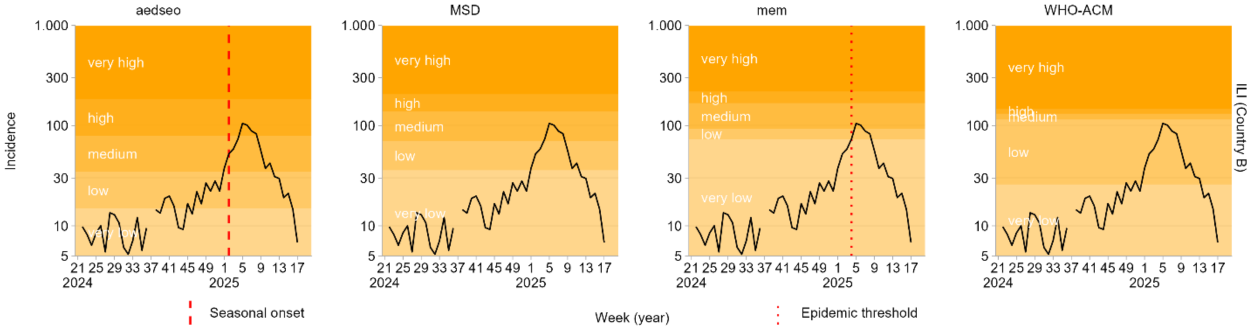

aedseo
MSD
mem
WHO-ACM
Incidence
ILI (Country B)
very high
high
medium
low
very low
Week (year)
Seasonal onset
Epidemic threshold


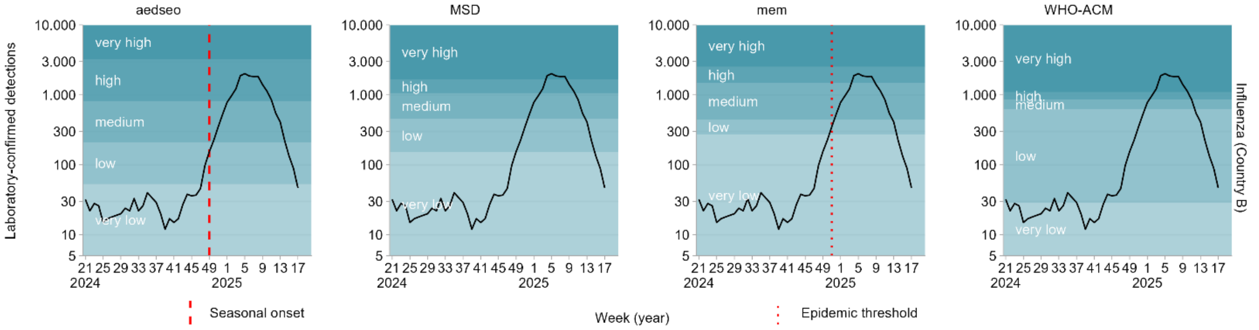

aedseo
MSD
mem
WHO-ACM
Laboratory-confirmed detections
Influenza (Country B)
very high
high
medium
low
very low
Week (year)
Seasonal onset
Epidemic threshold


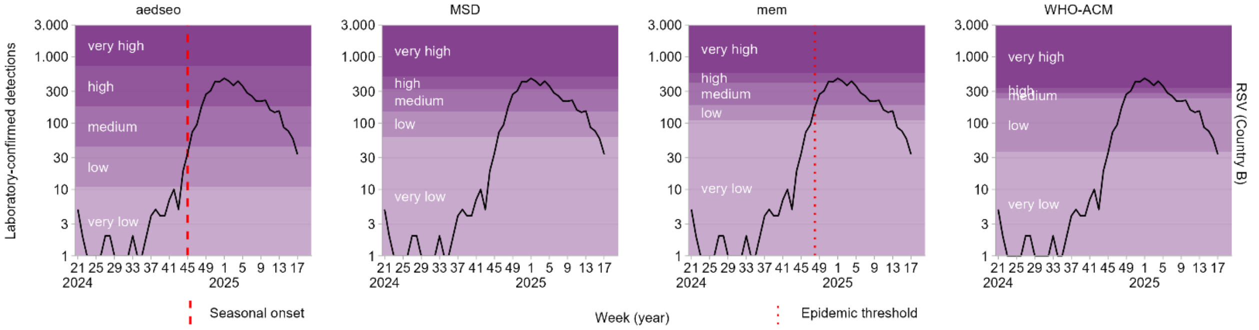

aedseo
MSD
mem
WHO-ACM
Laboratory-confirmed detections
RSV (Country B)
very high
high
medium
low
very low
Week (year)
Seasonal onset
Epidemic threshold

## Country C

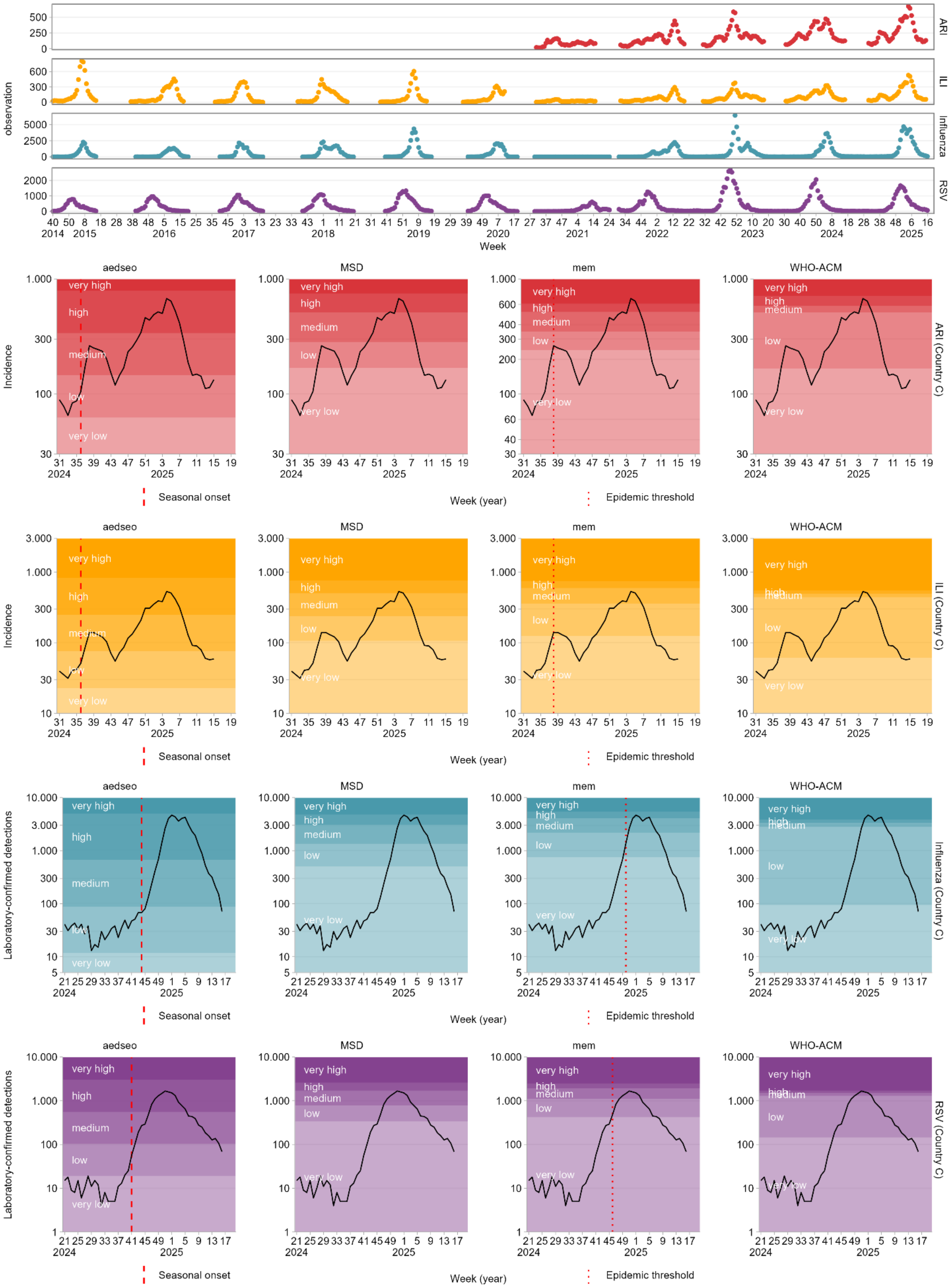

ARI
ILI
Influenza
RSV
observation
Week
aedseo
MSD
mem
WHO-ACM
very high
high
medium
low
very low
Incidence
Laboratory-confirmed detections
Week (year)
Seasonal onset
Epidemic threshold
ARI (Country C)
ILI (Country C)
Influenza (Country C)
RSV (Country C)

# Country D

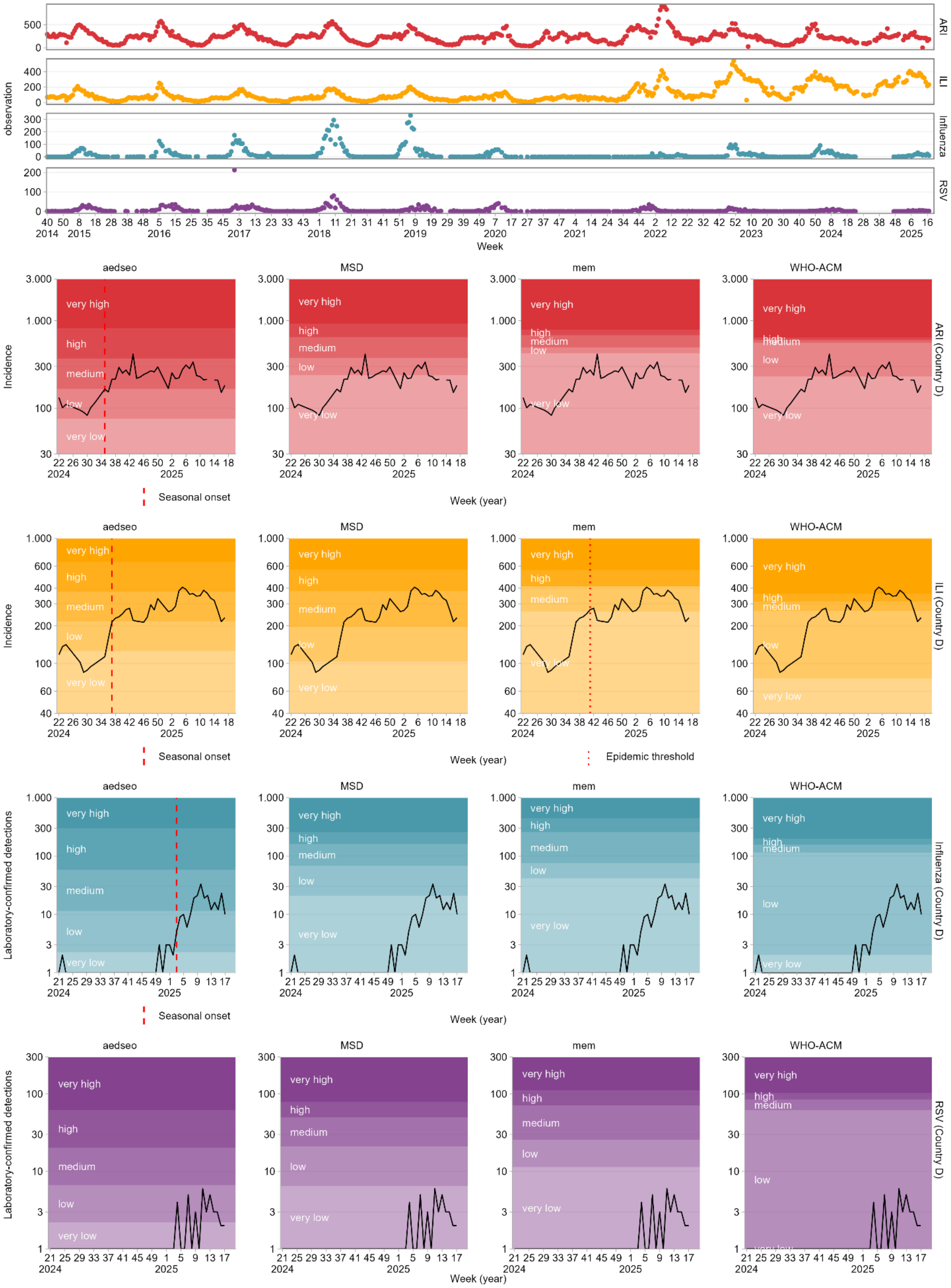

observation
ARI
ILI
Influenza
RSV
Week
aedseo
MSD
mem
WHO-ACM
Incidence
Laboratory-confirmed detections
very high
high
medium
low
very low
Seasonal onset
Epidemic threshold
Week (year)
ARI (Country D)
ILI (Country D)
Influenza (Country D)
RSV (Country D)
2024
2025

## Country E

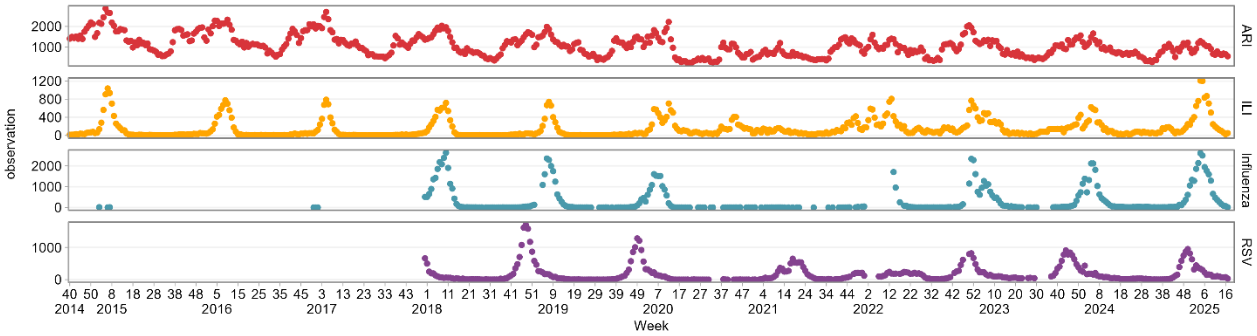
observation
ARI
ILI
Influenza
RSV
Week
2014 2015 2016 2017 2018 2019 2020 2021 2022 2023 2024 2025

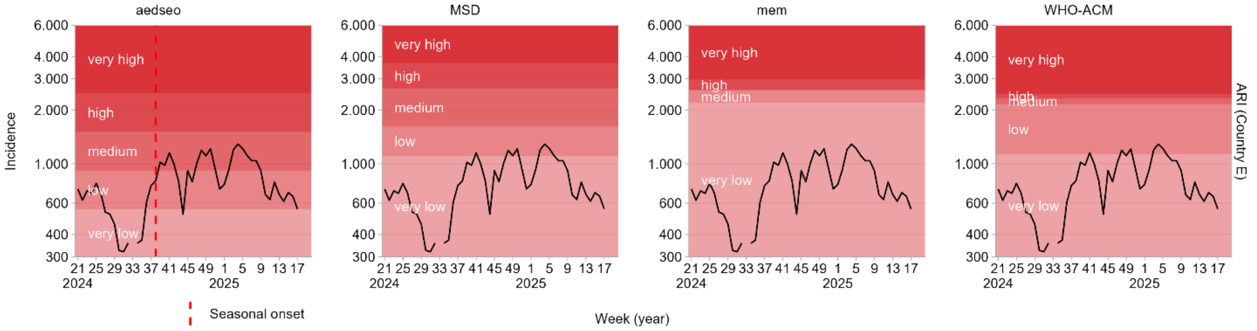
aedseo
MSD
mem
WHO-ACM
Incidence
very high
high
medium
low
very low
ARI (Country E)
Week (year)
Seasonal onset

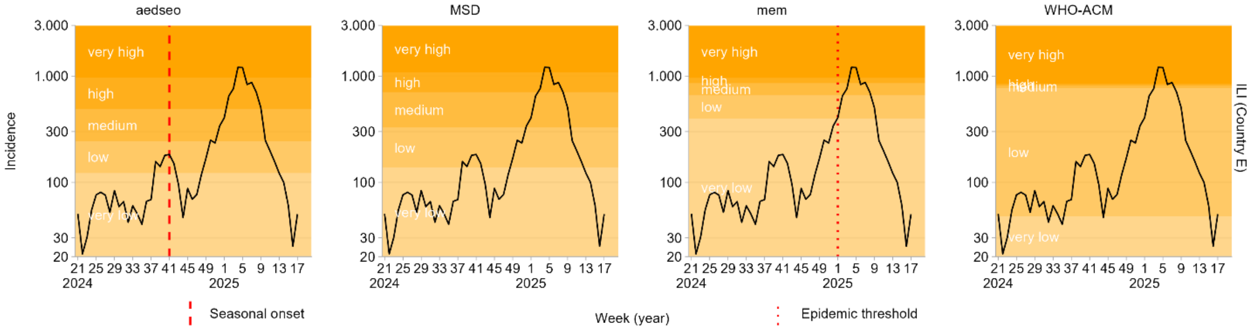
aedseo
MSD
mem
WHO-ACM
Incidence
very high
high
medium
low
very low
ILI (Country E)
Seasonal onset
Week (year)
Epidemic threshold

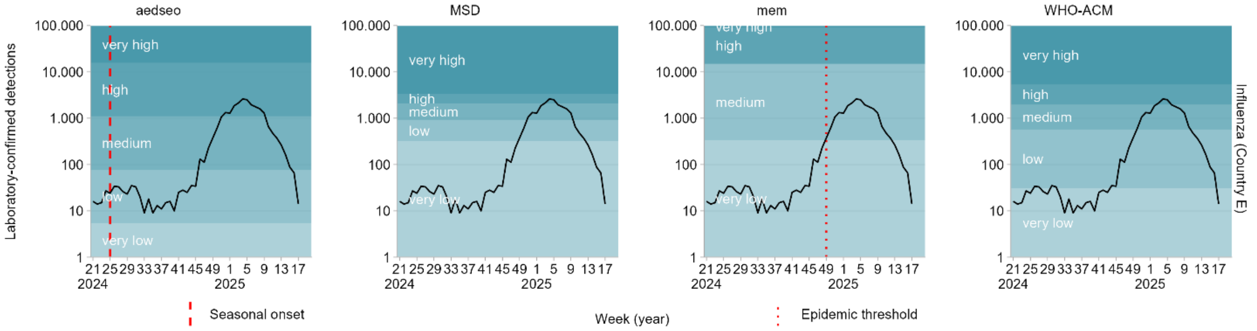
aedseo
MSD
mem
WHO-ACM
Laboratory-confirmed detections
very high
high
medium
low
very low
Influenza (Country E)
Seasonal onset
Week (year)
Epidemic threshold

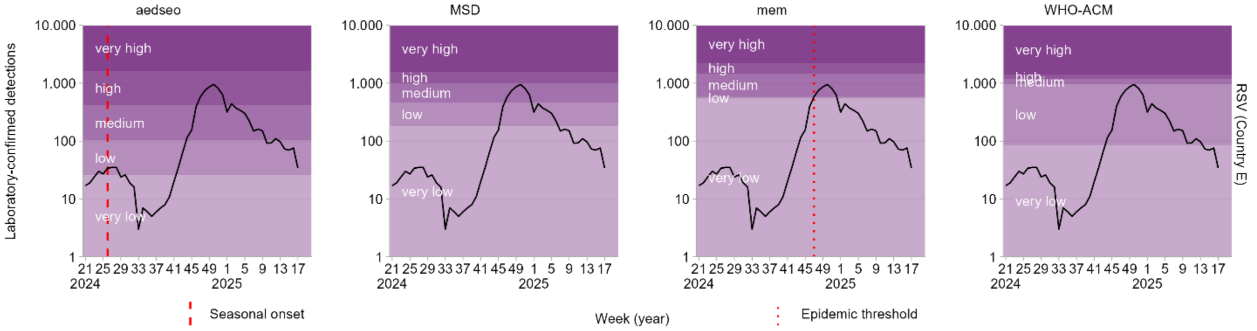
aedseo
MSD
mem
WHO-ACM
Laboratory-confirmed detections
very high
high
medium
low
very low
RSV (Country E)
Seasonal onset
Week (year)
Epidemic threshold

## Country F

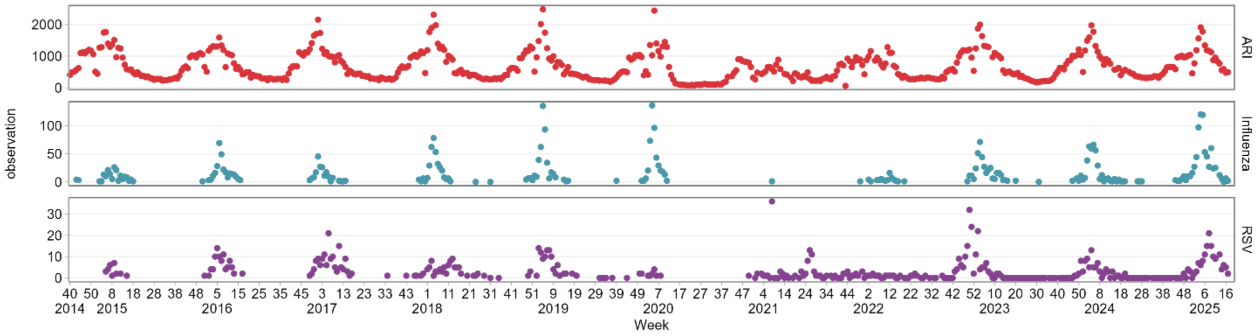

observation
ARI
Influenza
RSV
Week


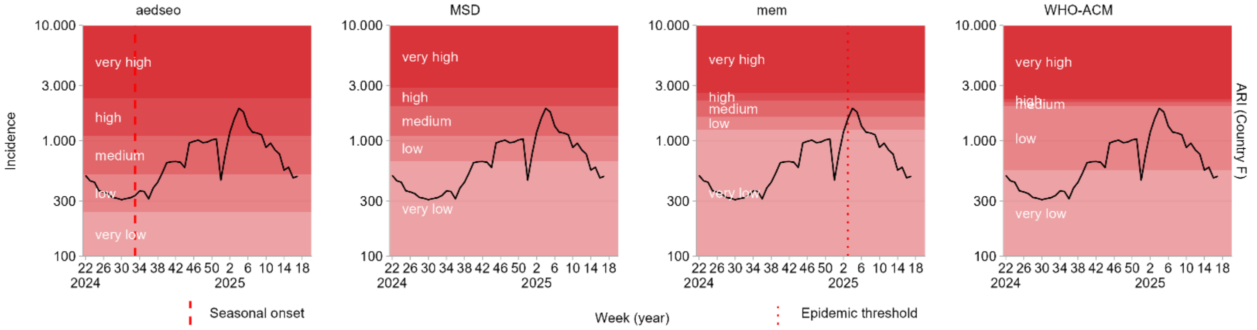

aedseo
MSD
mem
WHO-ACM
Incidence
ARI (Country F)
very high
high
medium
low
very low
Seasonal onset
Week (year)
Epidemic threshold


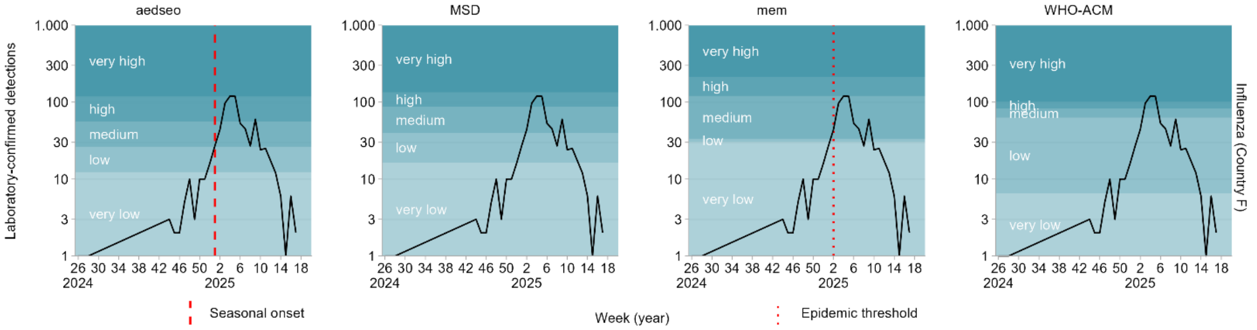

aedseo
MSD
mem
WHO-ACM
Laboratory-confirmed detections
Influenza (Country F)
very high
high
medium
low
very low
Seasonal onset
Week (year)
Epidemic threshold


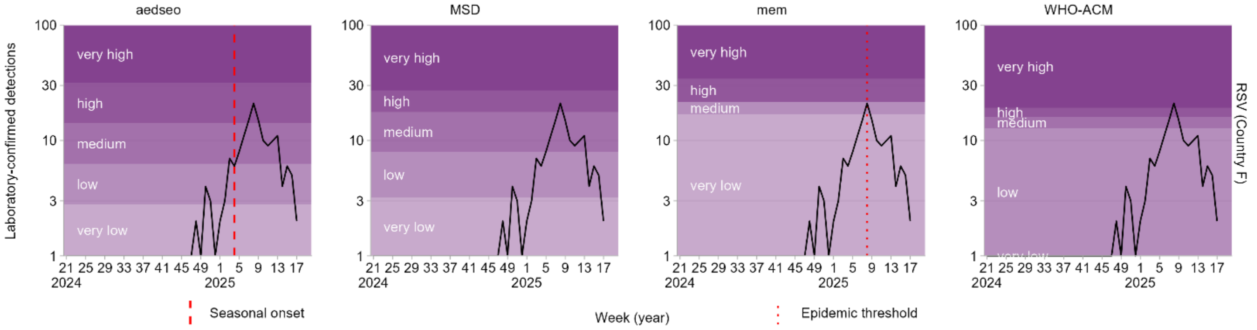

aedseo
MSD
mem
WHO-ACM
Laboratory-confirmed detections
RSV (Country F)
very high
high
medium
low
very low
Seasonal onset
Week (year)
Epidemic threshold

## Country G

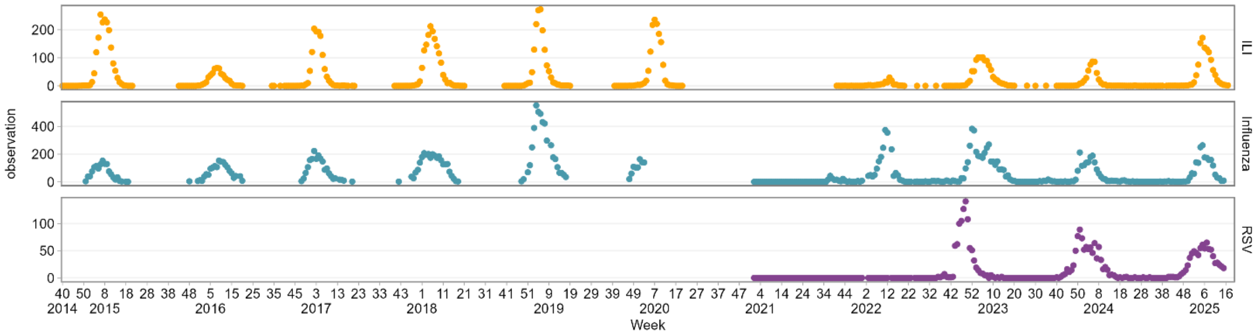

ILI
Influenza
RSV
observation
Week
2014
2015
2016
2017
2018
2019
2020
2021
2022
2023
2024
2025


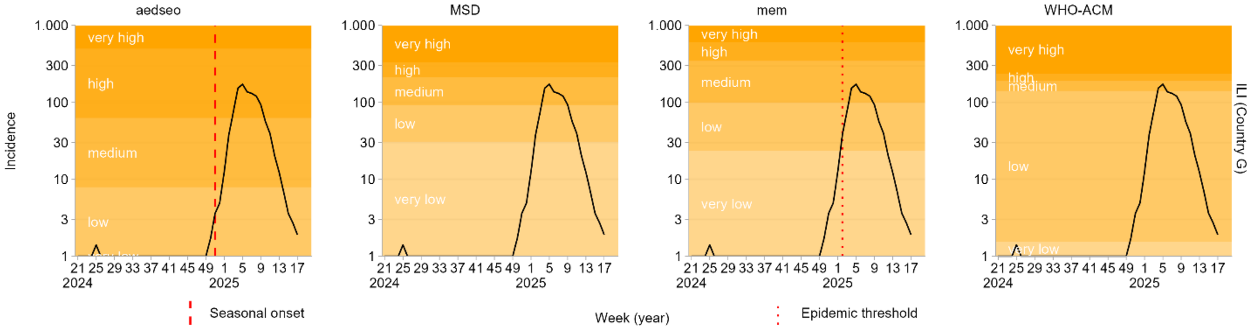

aedseo
MSD
mem
WHO-ACM
Incidence
very high
high
medium
low
very low
ILI (Country G)
Week (year)
Seasonal onset
Epidemic threshold


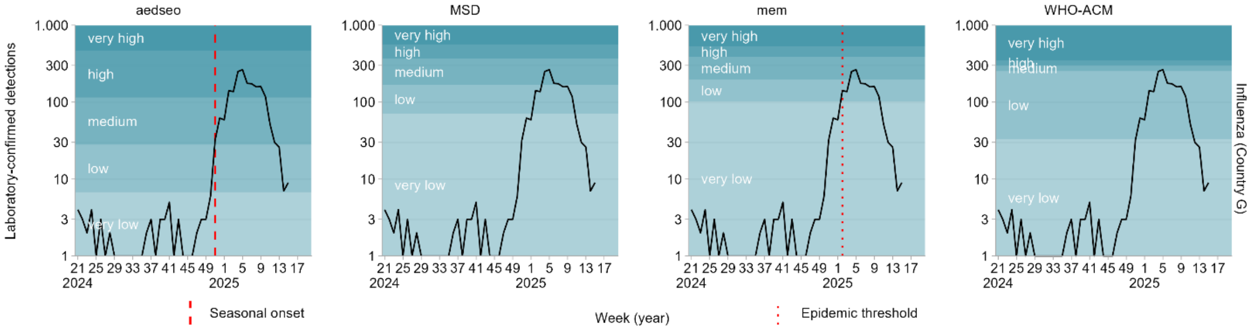

aedseo
MSD
mem
WHO-ACM
Laboratory-confirmed detections
very high
high
medium
low
very low
Influenza (Country G)
Week (year)
Seasonal onset
Epidemic threshold


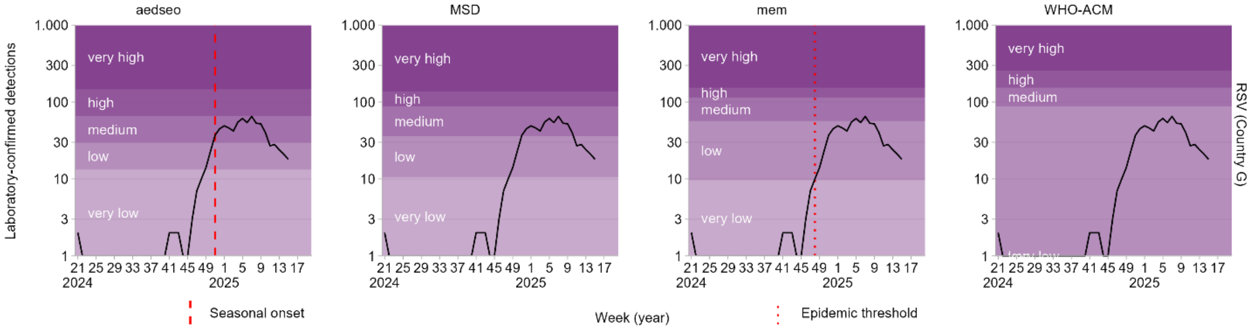

aedseo
MSD
mem
WHO-ACM
Laboratory-confirmed detections
very high
high
medium
low
very low
RSV (Country G)
Week (year)
Seasonal onset
Epidemic threshold

## Country H

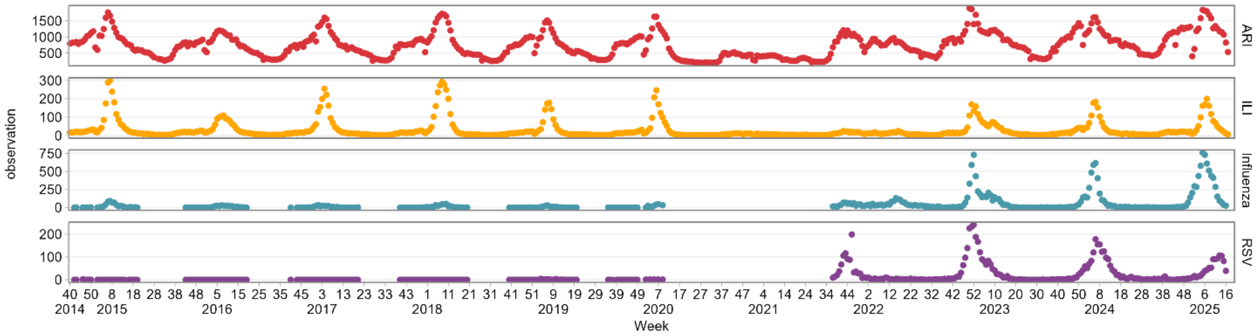
observation
ARI
ILI
Influenza
RSV
Week

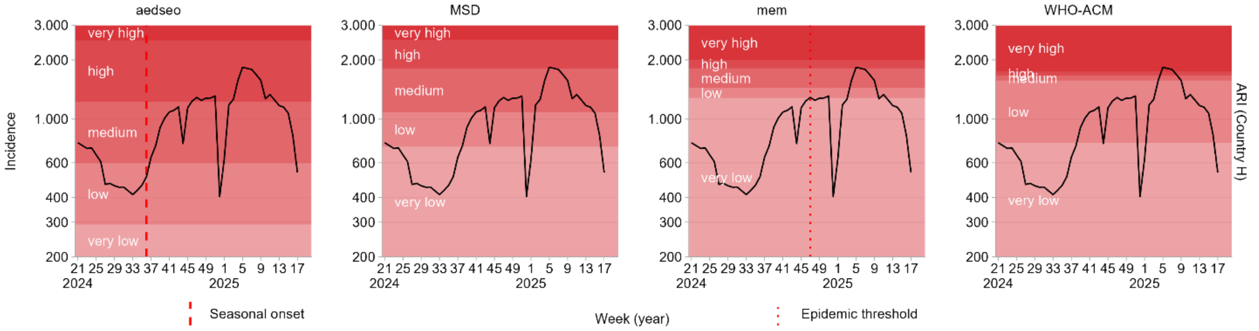
aedseo
MSD
mem
WHO-ACM
Incidence
ARI (Country H)
Seasonal onset
Week (year)
Epidemic threshold

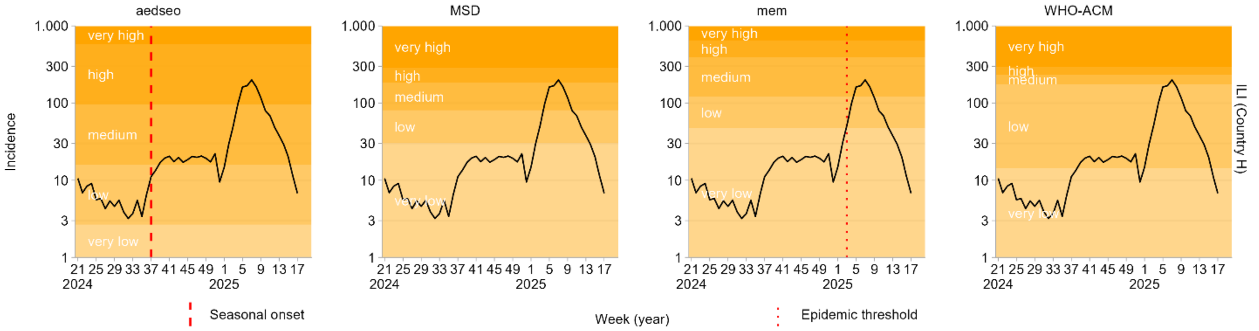
aedseo
MSD
mem
WHO-ACM
Incidence
ILI (Country H)
Seasonal onset
Week (year)
Epidemic threshold

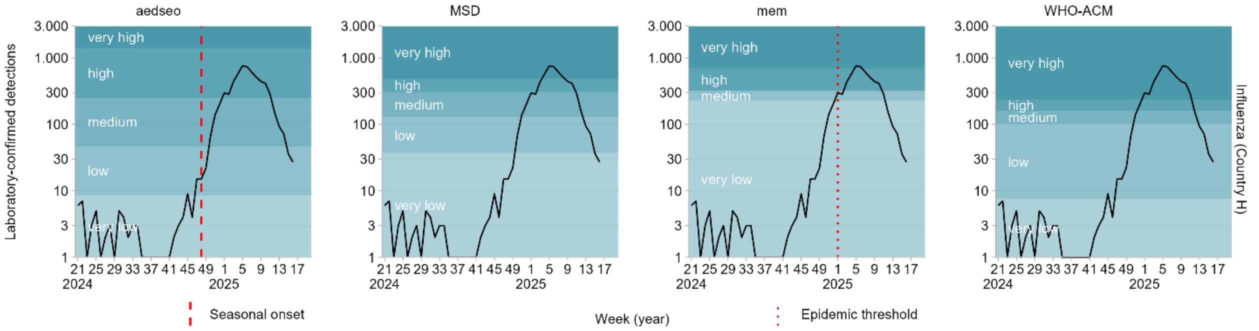
aedseo
MSD
mem
WHO-ACM
Laboratory-confirmed detections
Influenza (Country H)
Seasonal onset
Week (year)
Epidemic threshold

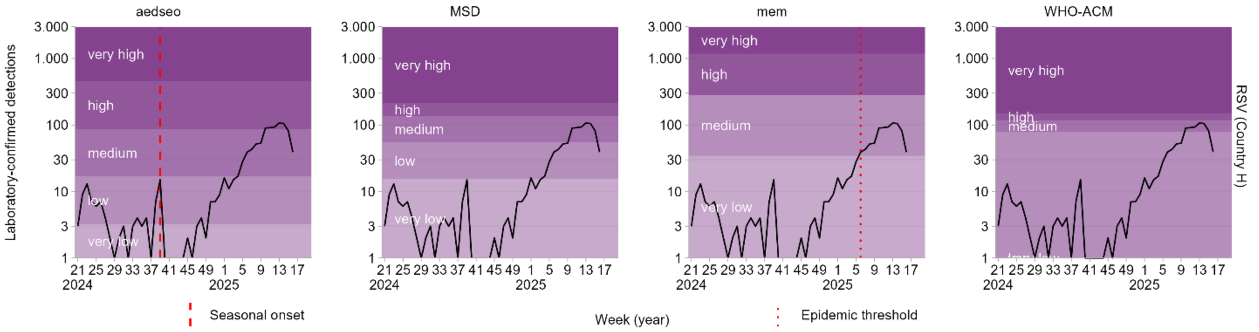
aedseo
MSD
mem
WHO-ACM
Laboratory-confirmed detections
RSV (Country H)
Seasonal onset
Week (year)
Epidemic threshold

## Country I

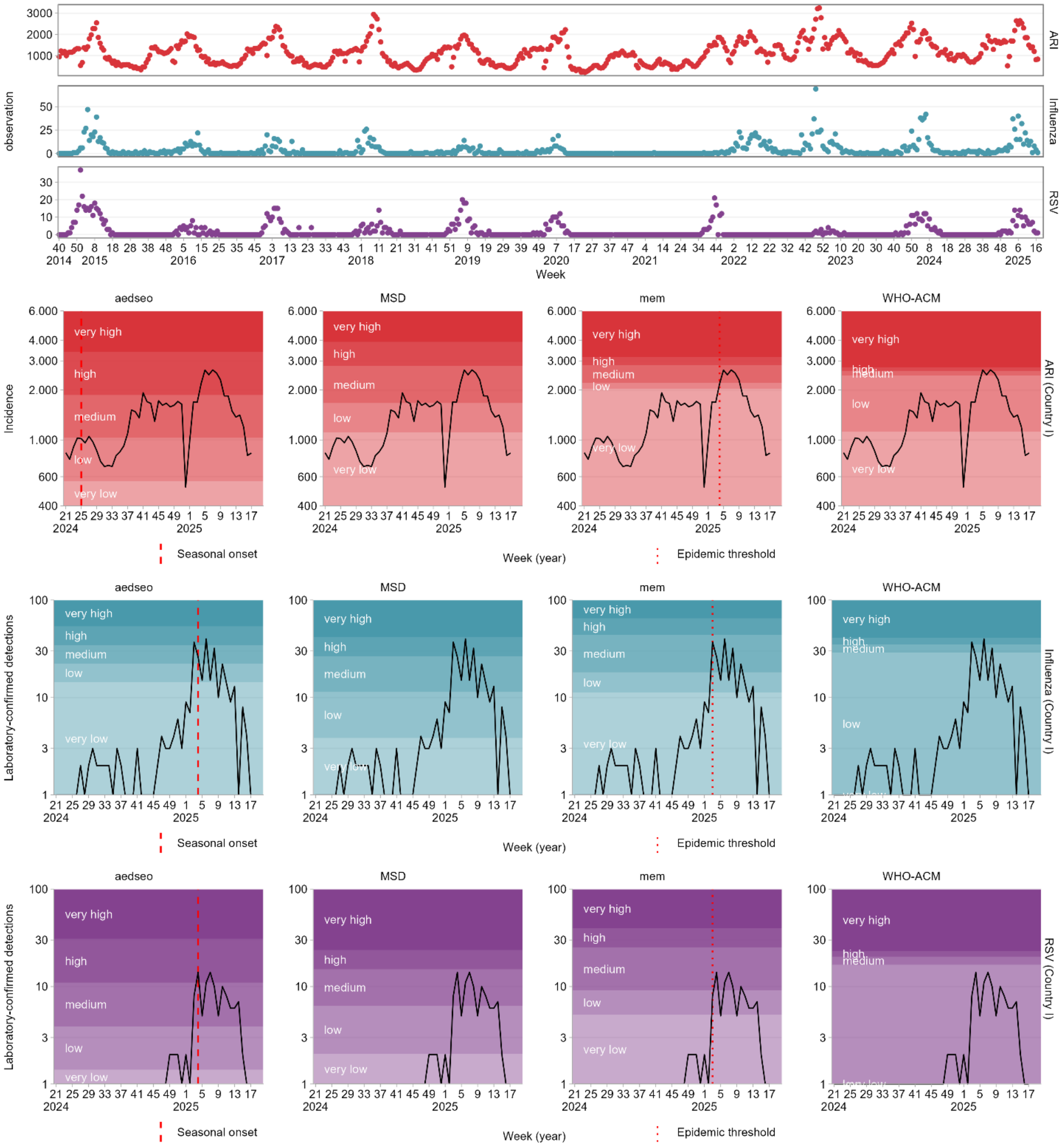
ARI
Influenza
RSV
observation
Week
aedseo
MSD
mem
WHO-ACM
Incidence
Laboratory-confirmed detections
ARI (Country I)
Influenza (Country I)
RSV (Country I)
very high
high
medium
low
very low
Week (year)
Seasonal onset
Epidemic threshold

## Country J

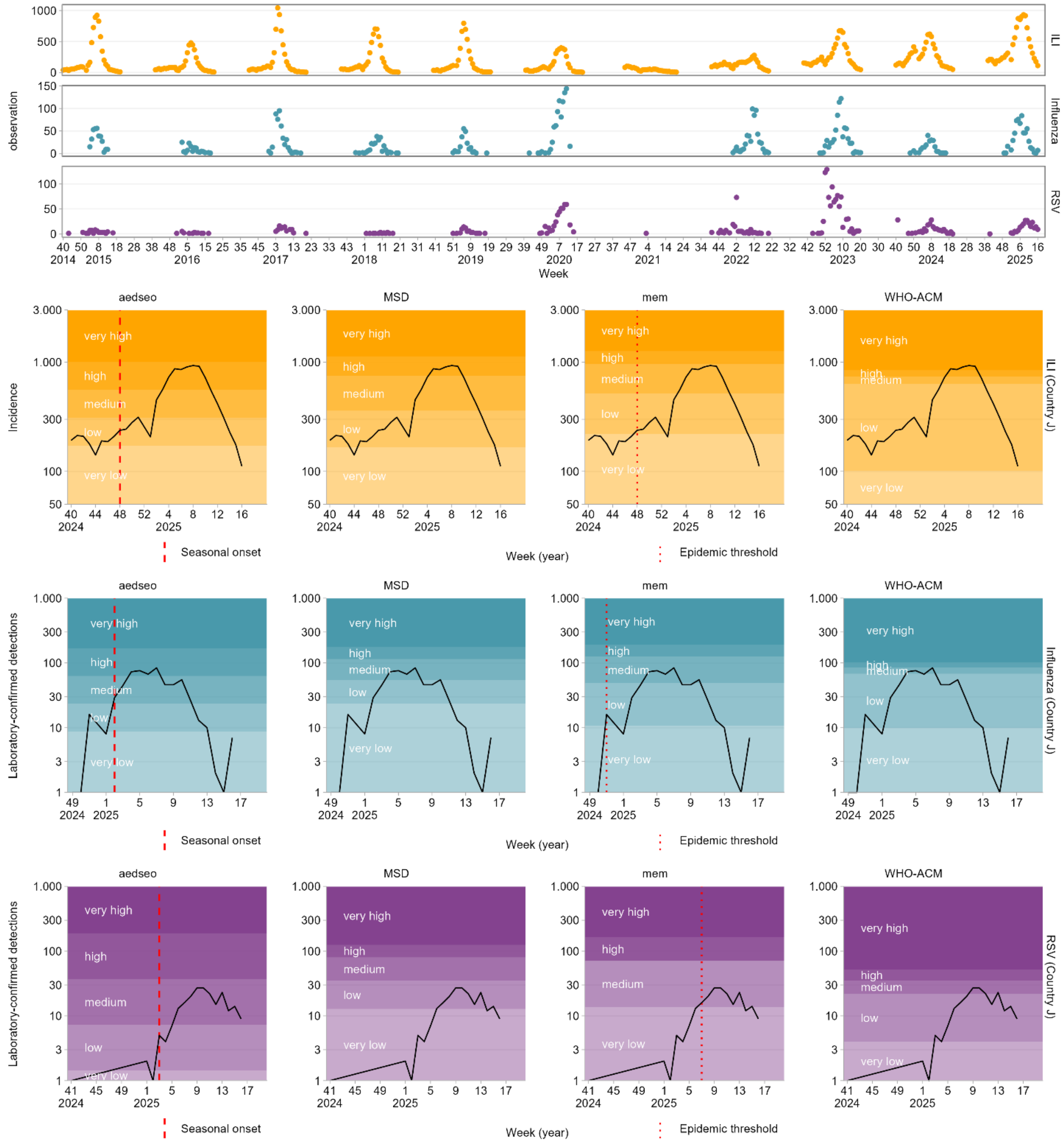

ILI
Influenza
RSV
observation
Week
aedseo
MSD
mem
WHO-ACM
very high
high
medium
low
very low
Incidence
ILI (Country J)
Influenza (Country J)
RSV (Country J)
Laboratory-confirmed detections
Week (year)
Seasonal onset
Epidemic threshold

## Country K

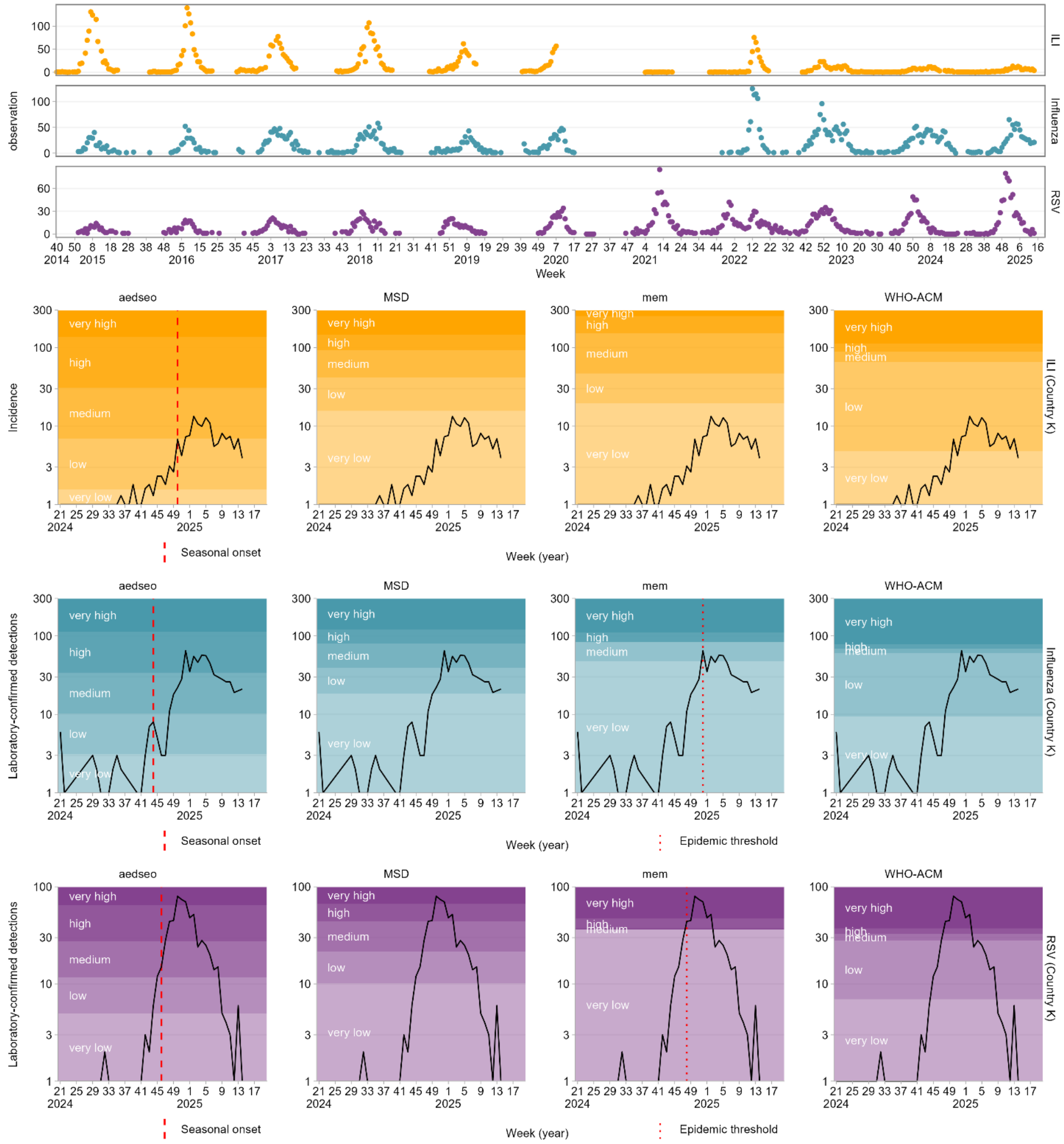

observation
ILI
Influenza
RSV
Week
aedseo
MSD
mem
WHO-ACM
Incidence
Laboratory-confirmed detections
Week (year)
Seasonal onset
Epidemic threshold
ILI (Country K)
Influenza (Country K)
RSV (Country K)
very high
high
medium
low
very low

## Country L

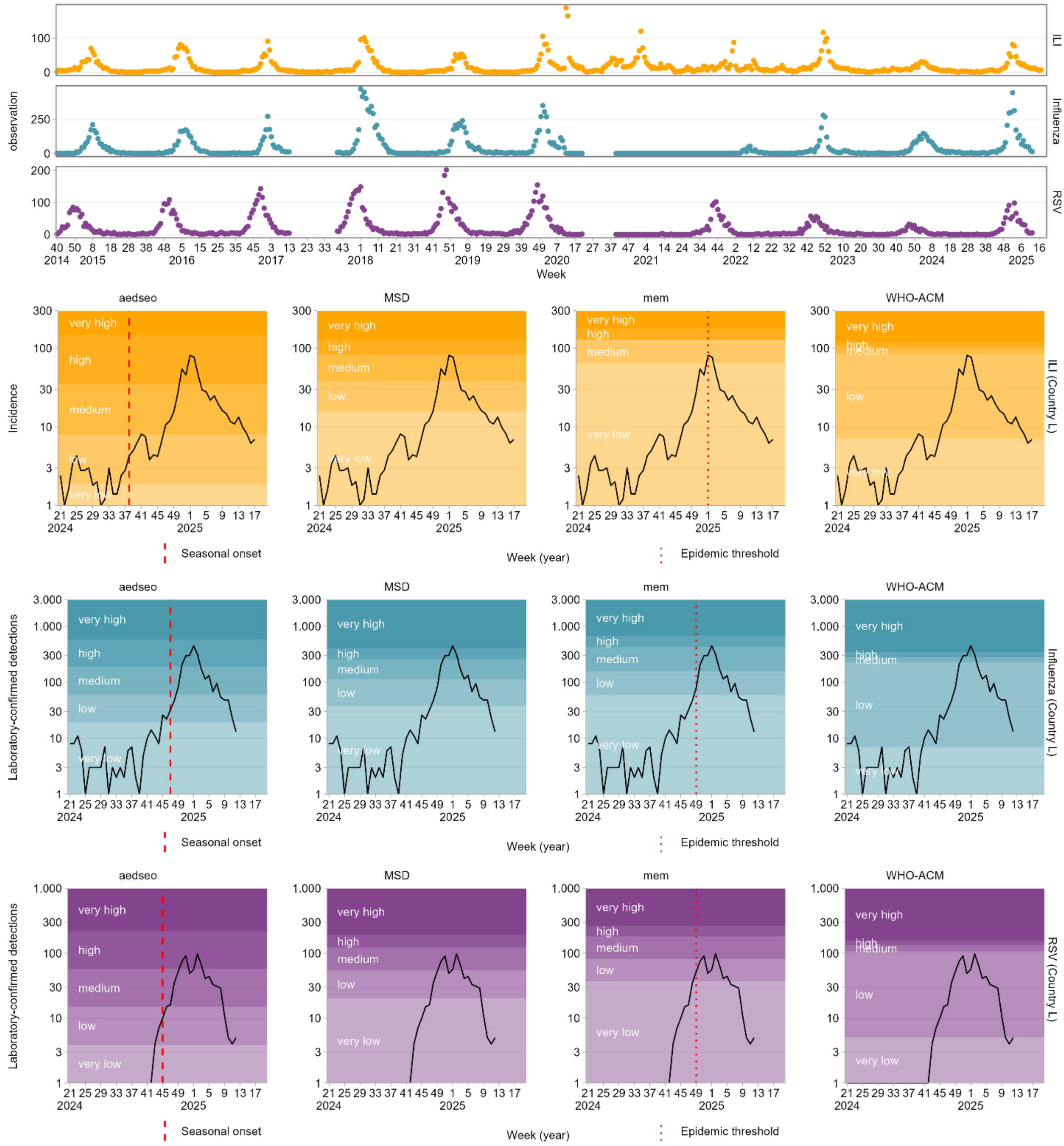

ILI
Influenza
RSV
observation
Week
aedseo
MSD
mem
WHO-ACM
Incidence
Laboratory-confirmed detections
very high
high
medium
low
very low
Seasonal onset
Week (year)
Epidemic threshold
ILI (Country L)
Influenza (Country L)
RSV (Country L)

## Country M

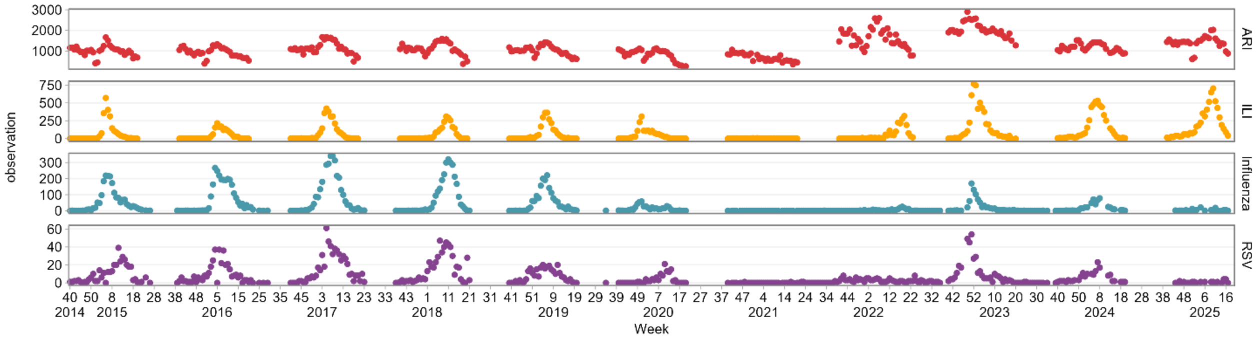
ARI
ILI
Influenza
RSV
observation
Week

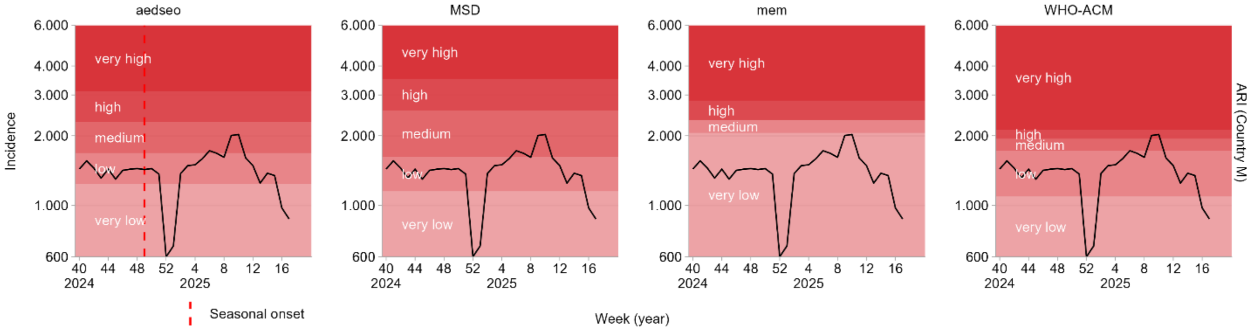
aedseo
MSD
mem
WHO-ACM
Incidence
very high
high
medium
low
very low
ARI (Country M)
Seasonal onset
Week (year)

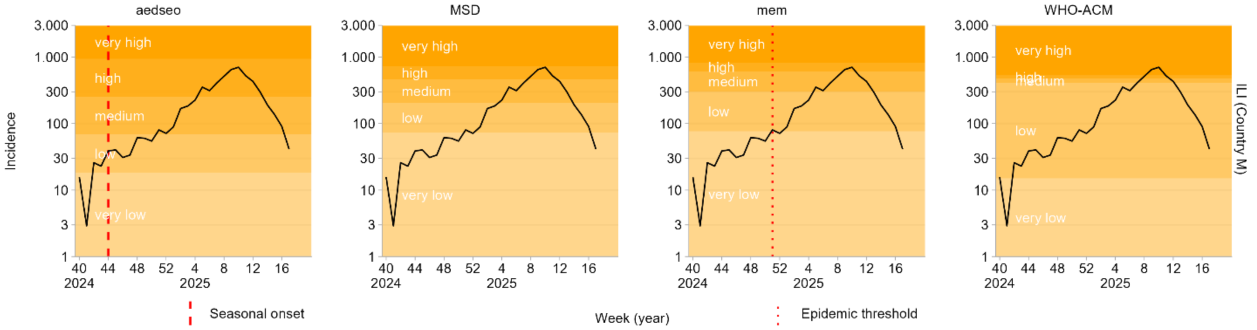
aedseo
MSD
mem
WHO-ACM
Incidence
very high
high
medium
low
very low
ILI (Country M)
Seasonal onset
Week (year)
Epidemic threshold

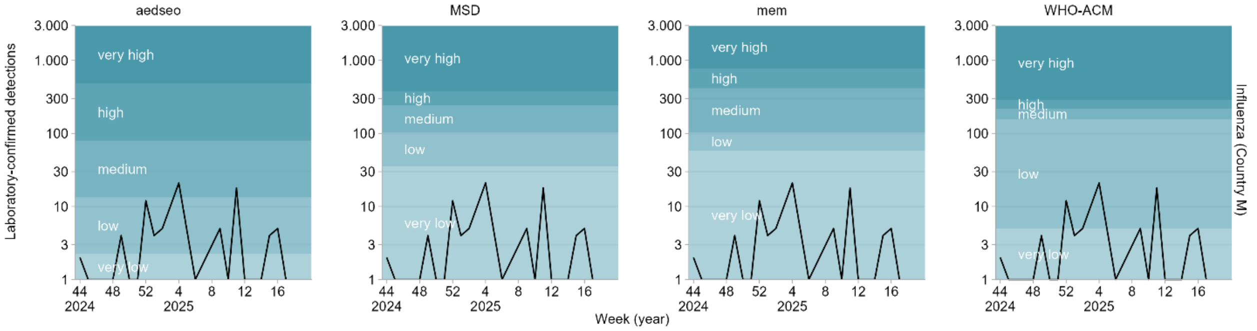
aedseo
MSD
mem
WHO-ACM
Laboratory-confirmed detections
very high
high
medium
low
very low
Influenza (Country M)
Week (year)

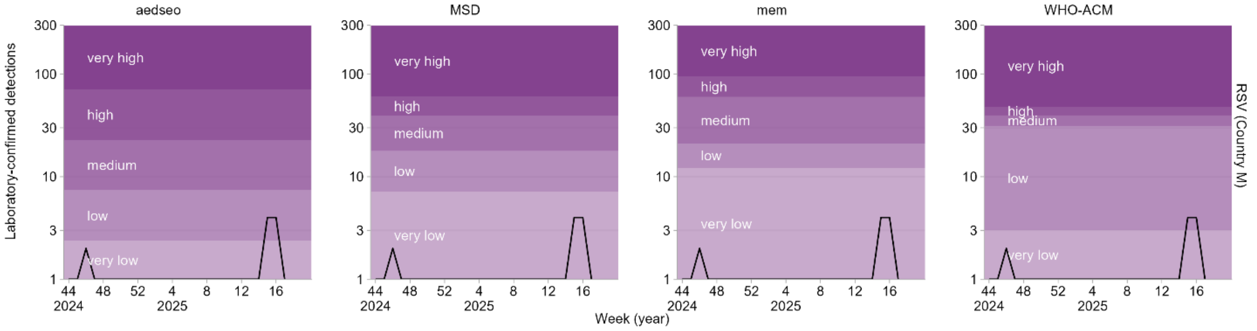
aedseo
MSD
mem
WHO-ACM
Laboratory-confirmed detections
very high
high
medium
low
very low
RSV (Country M)
Week (year)

## Country N

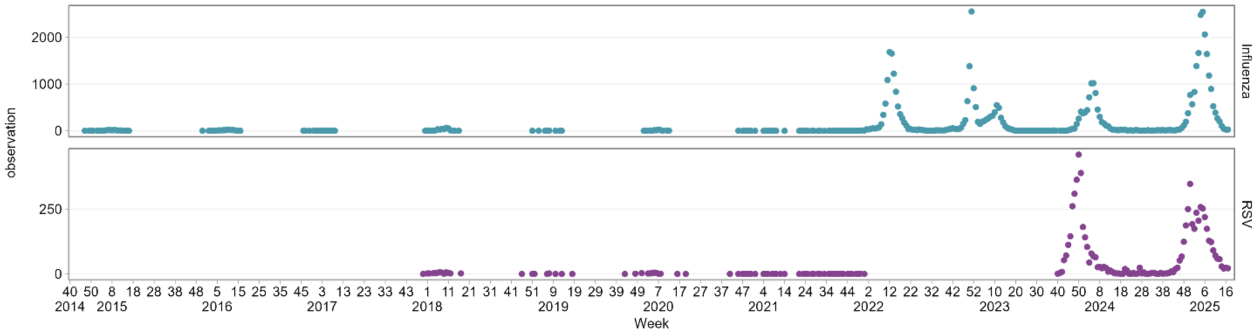

observation
Influenza
RSV
Week
2014
2015
2016
2017
2018
2019
2020
2021
2022
2023
2024
2025


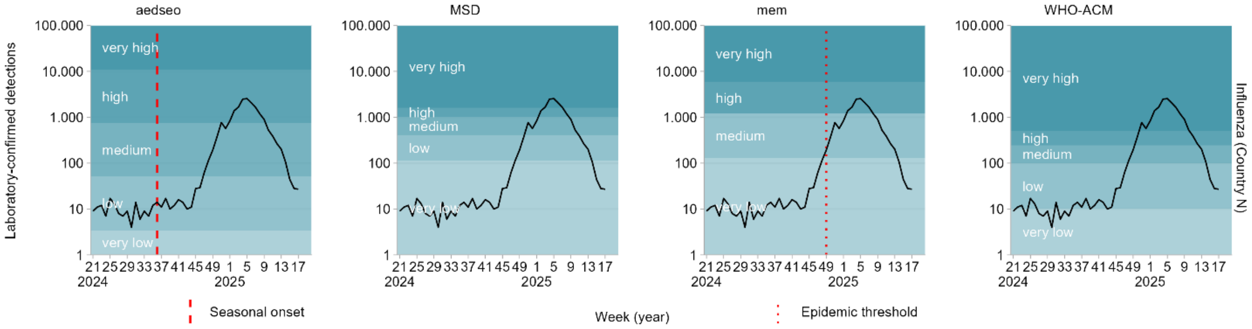

aedseo
MSD
mem
WHO-ACM
Laboratory-confirmed detections
Influenza (Country N)
very high
high
medium
low
very low
Seasonal onset
Week (year)
Epidemic threshold


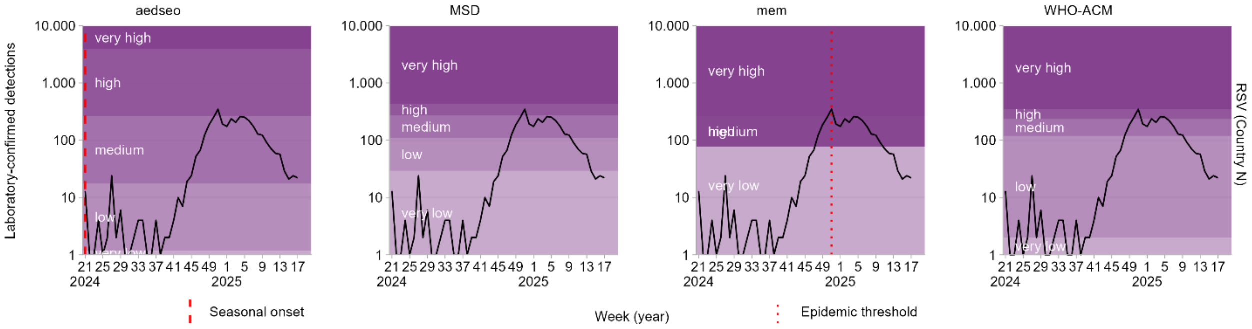

aedseo
MSD
mem
WHO-ACM
Laboratory-confirmed detections
RSV (Country N)
very high
high
medium
low
very low
Seasonal onset
Week (year)
Epidemic threshold

## Country O

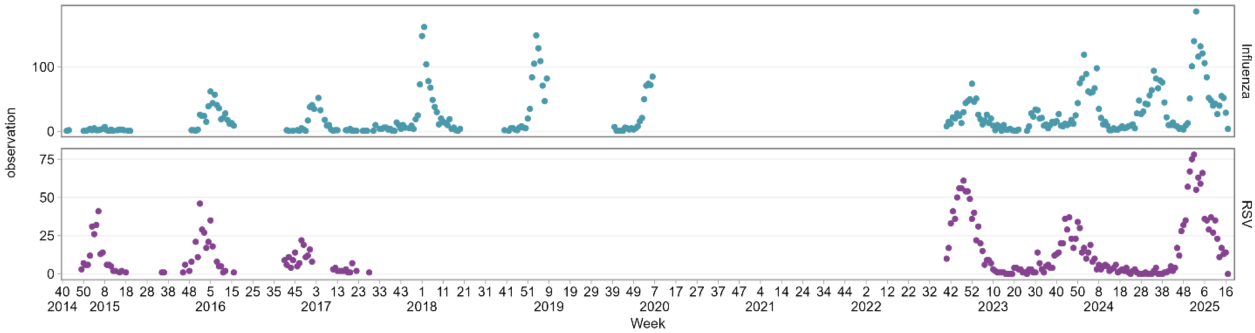

observation
Influenza
RSV
Week
2014
2015
2016
2017
2018
2019
2020
2021
2022
2023
2024
2025


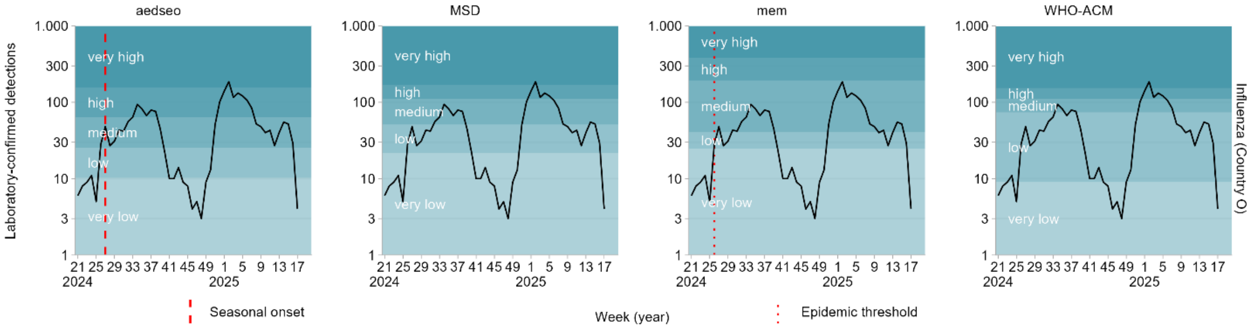

aedseo
MSD
mem
WHO-ACM
Laboratory-confirmed detections
Influenza (Country O)
very high
high
medium
low
very low
Week (year)
Seasonal onset
Epidemic threshold


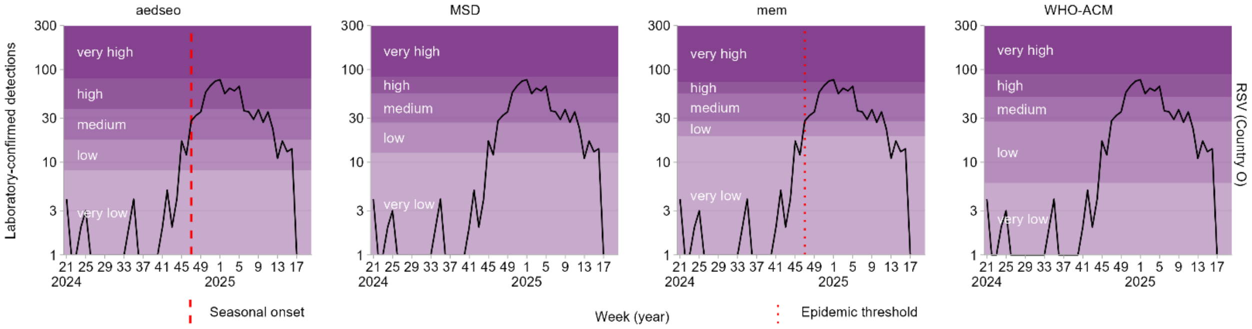

aedseo
MSD
mem
WHO-ACM
Laboratory-confirmed detections
RSV (Country O)
very high
high
medium
low
very low
Week (year)
Seasonal onset
Epidemic threshold

# Country P

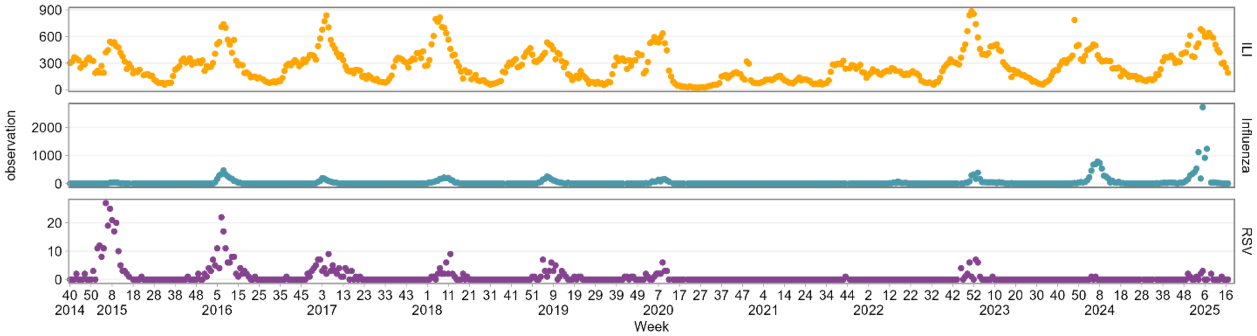
observation
ILI
Influenza
RSV
Week

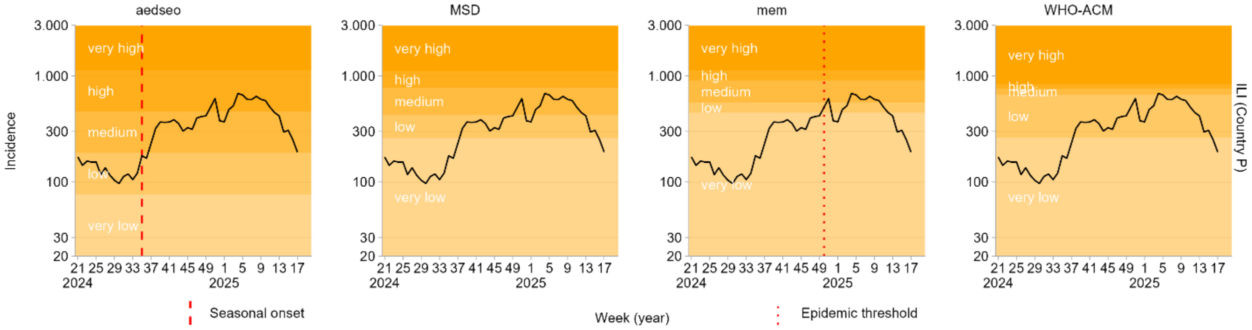
aedseo
MSD
mem
WHO-ACM
Incidence
ILI (Country P)
very high
high
medium
low
very low
Week (year)
Seasonal onset
Epidemic threshold

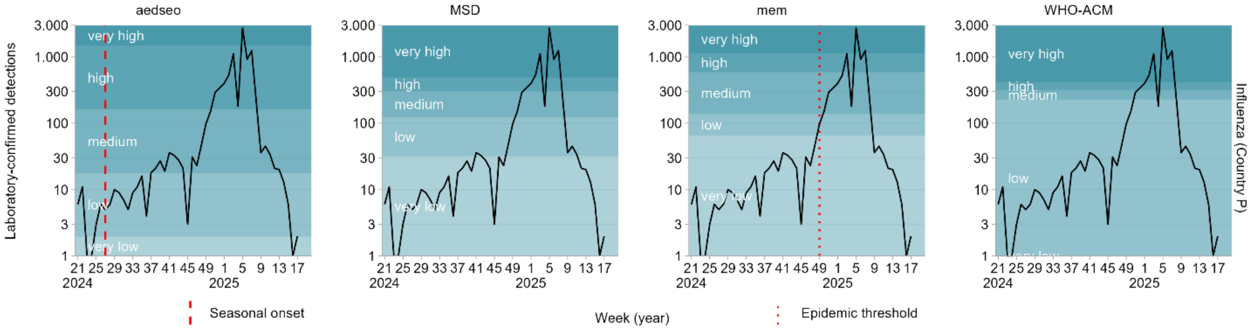
aedseo
MSD
mem
WHO-ACM
Laboratory-confirmed detections
Influenza (Country P)
very high
high
medium
low
very low
Week (year)
Seasonal onset
Epidemic threshold

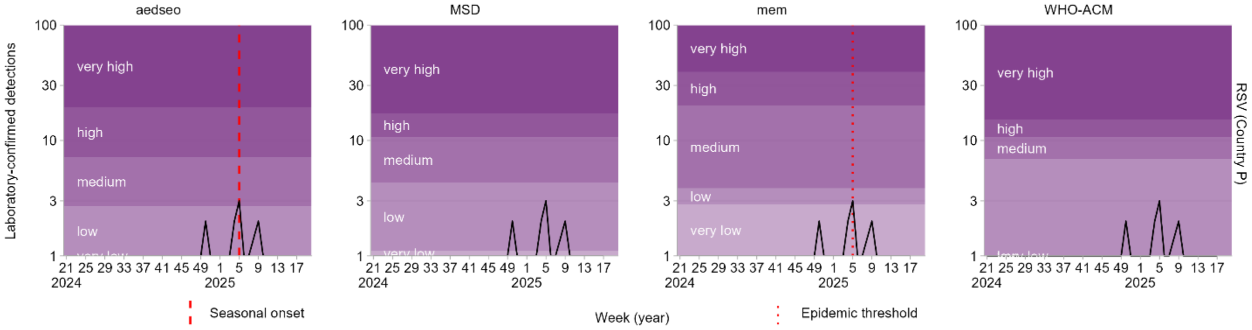
aedseo
MSD
mem
WHO-ACM
Laboratory-confirmed detections
RSV (Country P)
very high
high
medium
low
very low
Week (year)
Seasonal onset
Epidemic threshold

## Country Q

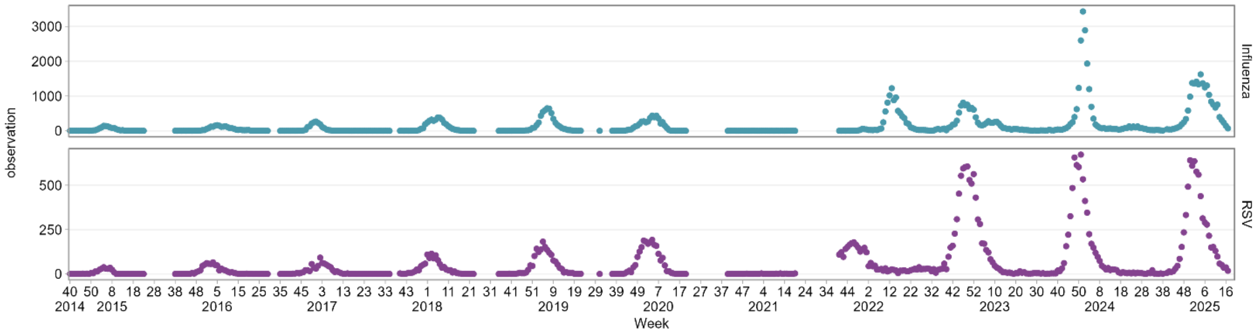

observation
Influenza
RSV
Week
3000
2000
1000
0
500
250
0
2014
2015
2016
2017
2018
2019
2020
2021
2022
2023
2024
2025


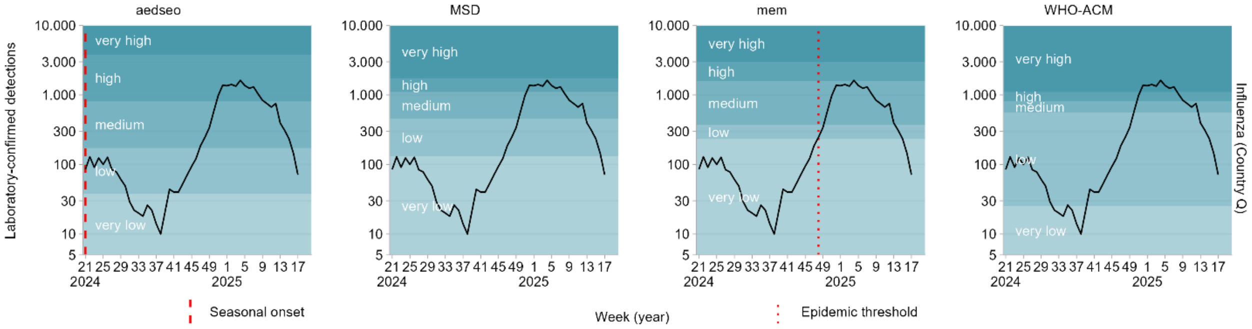

aedseo
MSD
mem
WHO-ACM
Laboratory-confirmed detections
Influenza (Country Q)
very high
high
medium
low
very low
10.000
3.000
1.000
300
100
30
10
5
21 25 29 33 37 41 45 49 1 5 9 13 17
2024
2025
Seasonal onset
Week (year)
Epidemic threshold


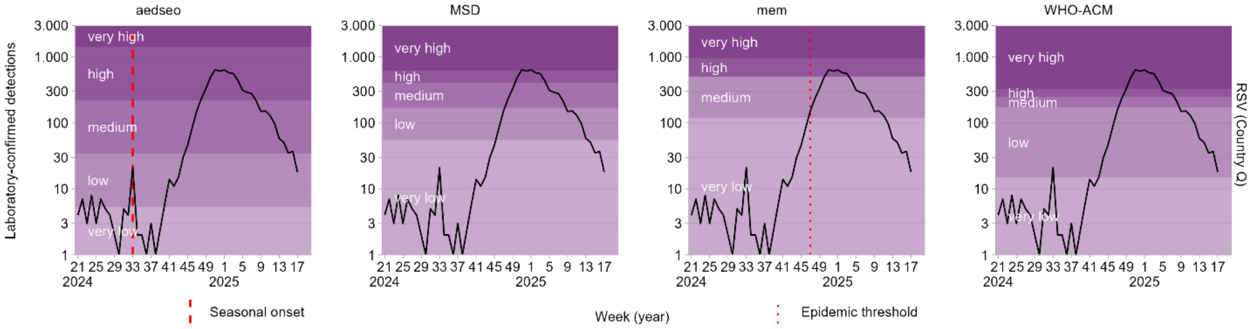

aedseo
MSD
mem
WHO-ACM
Laboratory-confirmed detections
RSV (Country Q)
very high
high
medium
low
very low
3.000
1.000
300
100
30
10
3
1
21 25 29 33 37 41 45 49 1 5 9 13 17
2024
2025
Seasonal onset
Week (year)
Epidemic threshold

## Country R

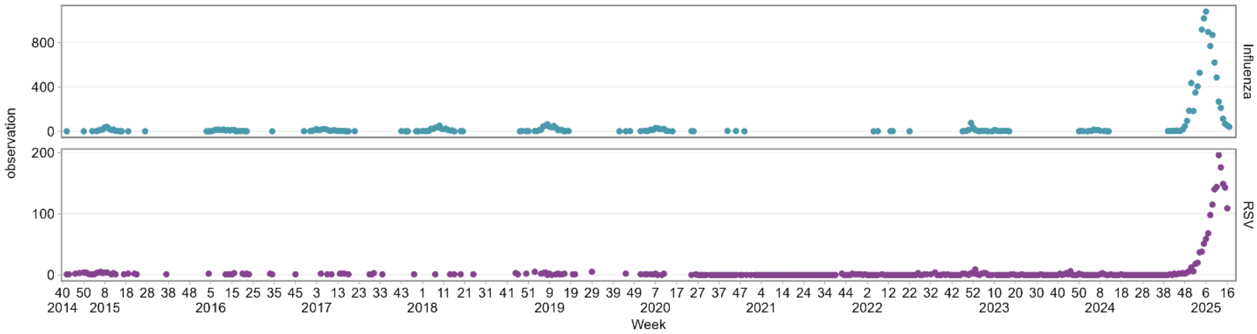

observation
Influenza
RSV
Week
2014 2015 2016 2017 2018 2019 2020 2021 2022 2023 2024 2025


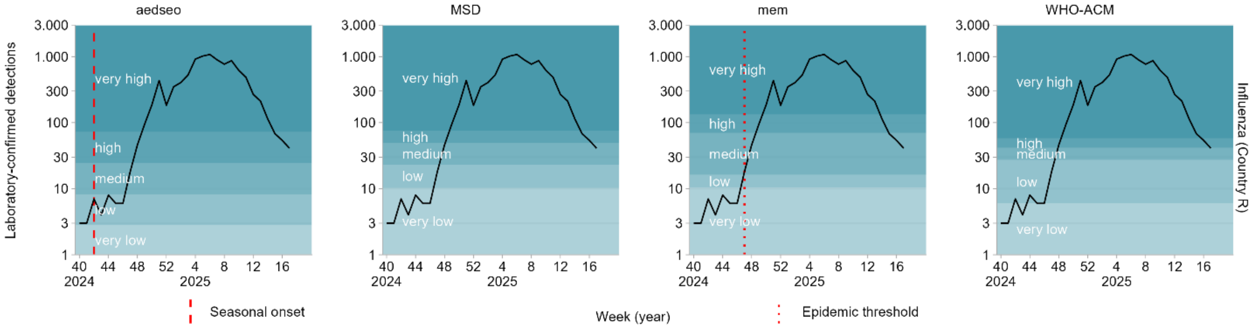

aedseo
MSD
mem
WHO-ACM
Laboratory-confirmed detections
Influenza (Country R)
very high
high
medium
low
very low
Week (year)
Seasonal onset
Epidemic threshold


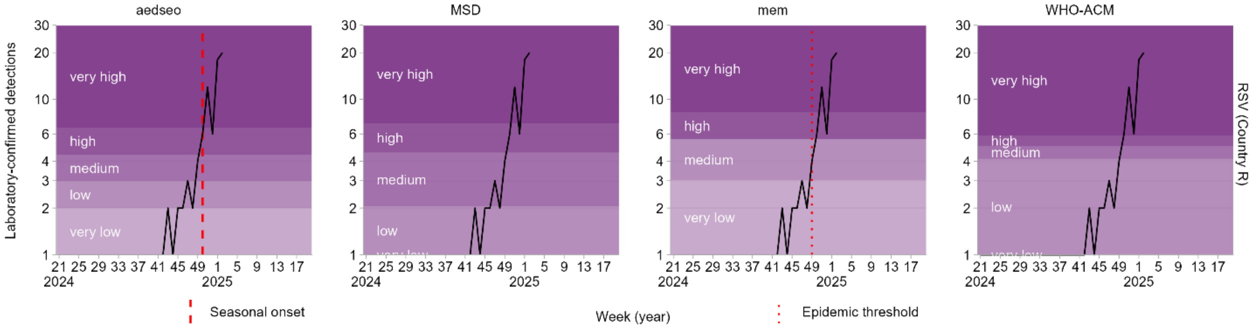

aedseo
MSD
mem
WHO-ACM
Laboratory-confirmed detections
RSV (Country R)
very high
high
medium
low
very low
Week (year)
Seasonal onset
Epidemic threshold

## Country S

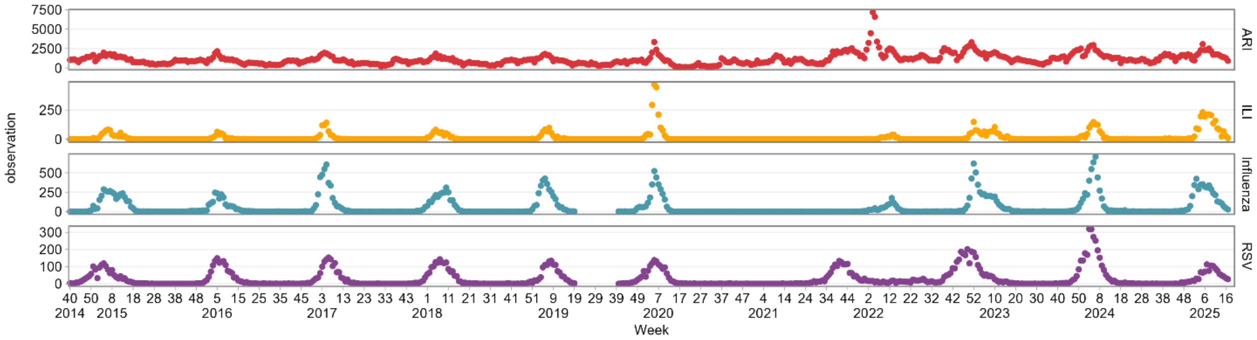
observation
ARI
ILI
Influenza
RSV
Week

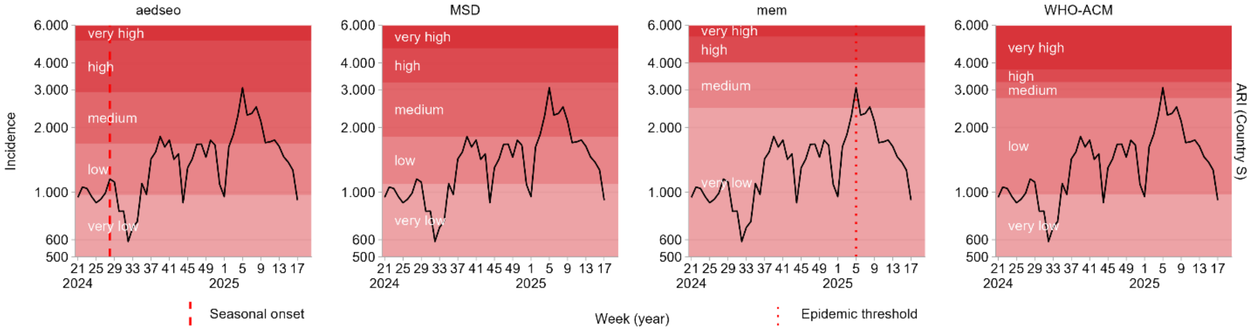
aedseo
MSD
mem
WHO-ACM
Incidence
ARI (Country S)
very high
high
medium
low
very low
Seasonal onset
Week (year)
Epidemic threshold

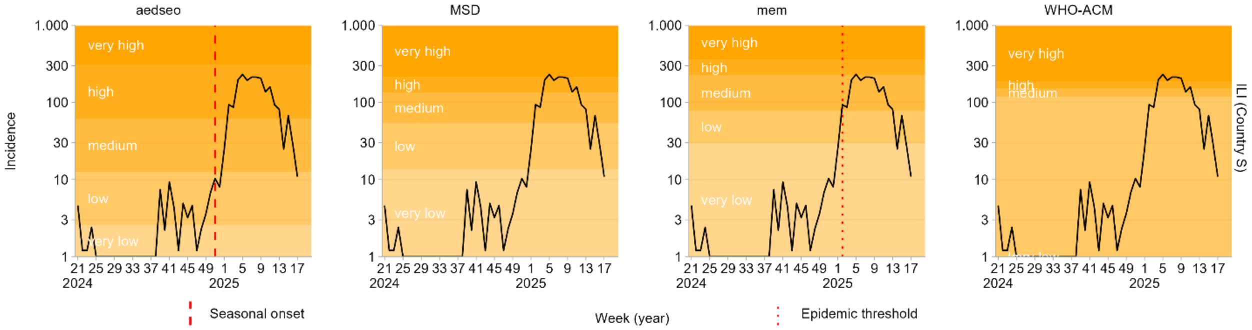
aedseo
MSD
mem
WHO-ACM
Incidence
ILI (Country S)
very high
high
medium
low
very low
Seasonal onset
Week (year)
Epidemic threshold

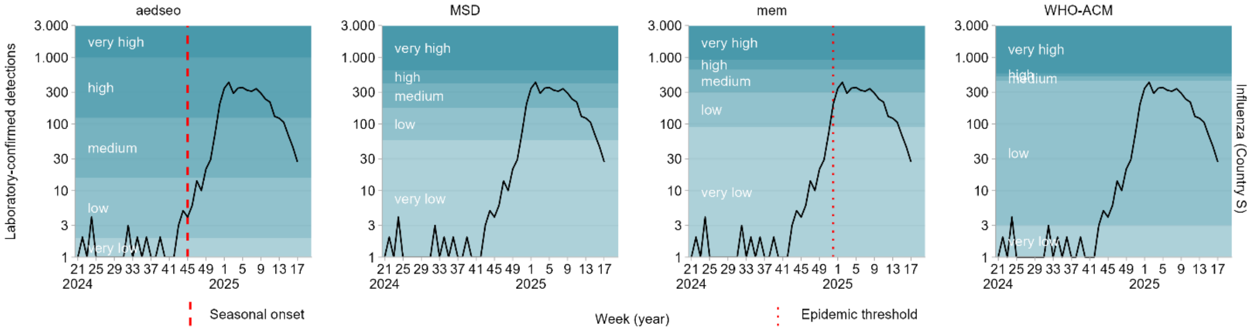
aedseo
MSD
mem
WHO-ACM
Laboratory-confirmed detections
Influenza (Country S)
very high
high
medium
low
very low
Seasonal onset
Week (year)
Epidemic threshold

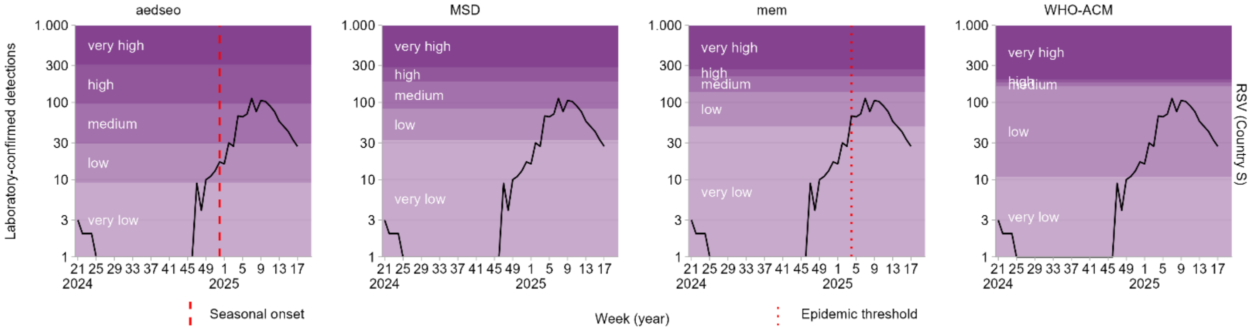
aedseo
MSD
mem
WHO-ACM
Laboratory-confirmed detections
RSV (Country S)
very high
high
medium
low
very low
Seasonal onset
Week (year)
Epidemic threshold

# Country T

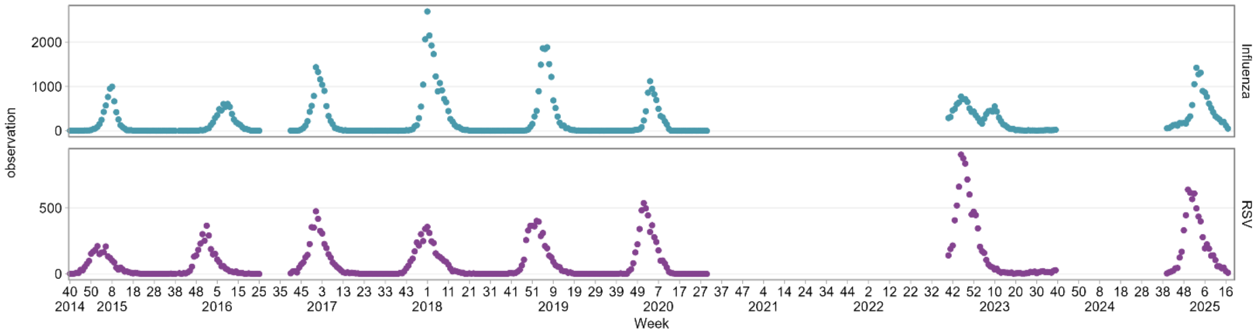
observation
Influenza
RSV
2000
1000
0
500
0
40 50 8 18 28 38 48 5 15 25 35 45 3 13 23 33 43 1 11 21 31 41 51 9 19 29 39 49 7 17 27 37 47 4 14 24 34 44 2 12 22 32 42 52 10 20 30 40 50 8 18 28 38 48 6 16
2014 2015 2016 2017 2018 2019 2020 2021 2022 2023 2024 2025
Week

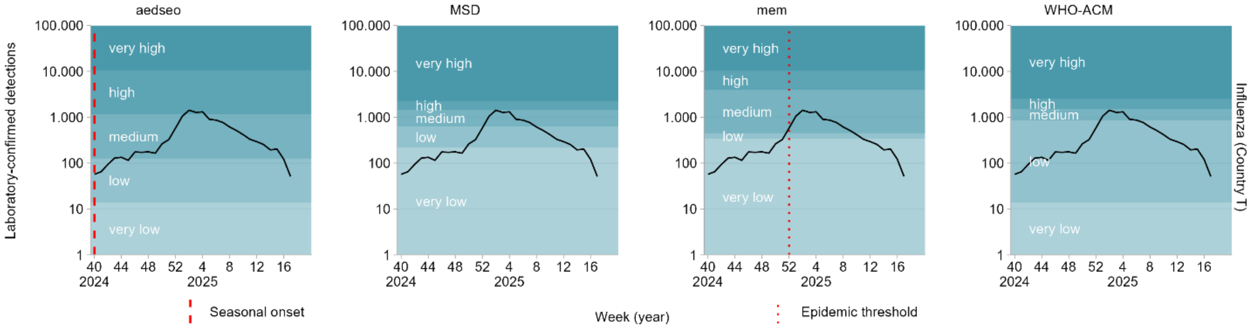
aedseo
MSD
mem
WHO-ACM
Laboratory-confirmed detections
Influenza (Country T)
100.000
10.000
1.000
100
10
1
very high
high
medium
low
very low
40 44 48 52 4 8 12 16
2024 2025
Seasonal onset
Week (year)
Epidemic threshold

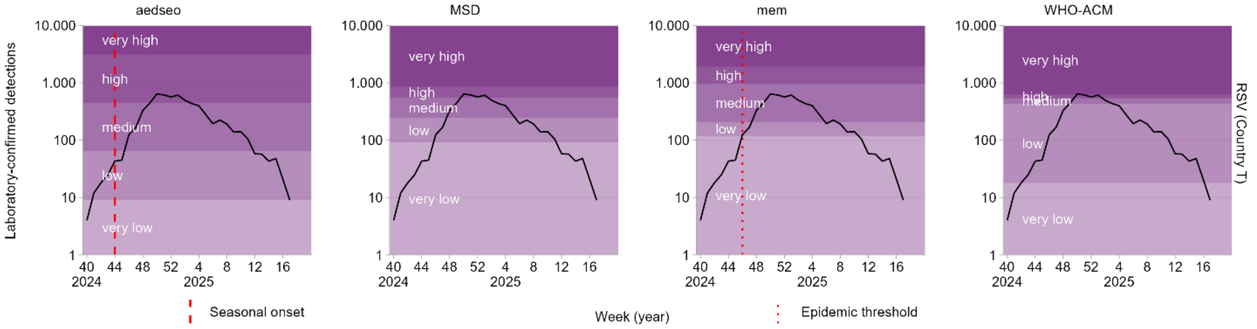
aedseo
MSD
mem
WHO-ACM
Laboratory-confirmed detections
RSV (Country T)
10.000
1.000
100
10
1
very high
high
medium
low
very low
40 44 48 52 4 8 12 16
2024 2025
Seasonal onset
Week (year)
Epidemic threshold

## Country U

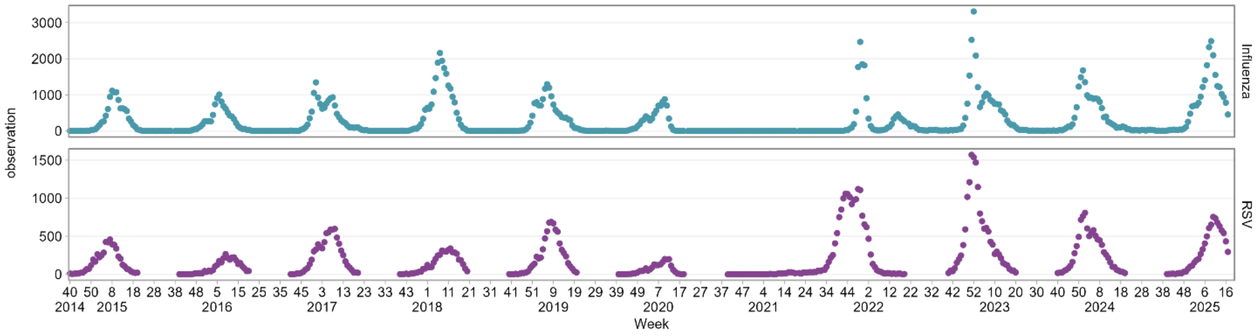
observation
Influenza
RSV
Week
2014
2015
2016
2017
2018
2019
2020
2021
2022
2023
2024
2025

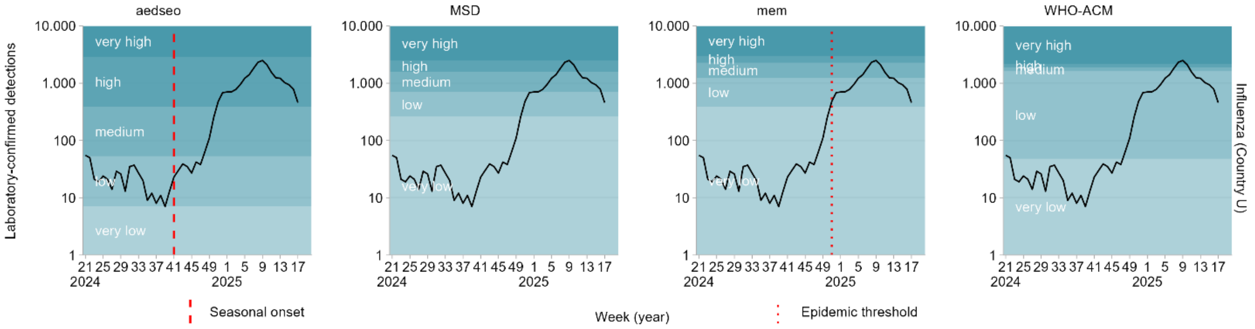
aedseo
MSD
mem
WHO-ACM
Laboratory-confirmed detections
Influenza (Country U)
very high
high
medium
low
very low
Week (year)
Seasonal onset
Epidemic threshold

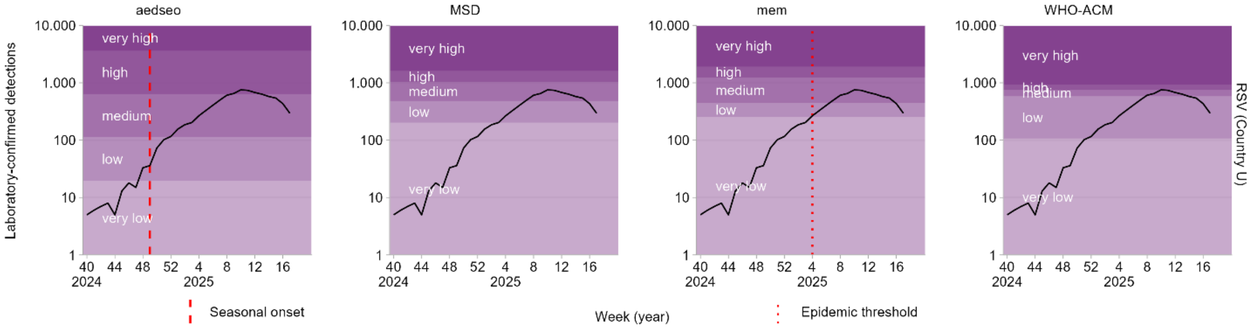
aedseo
MSD
mem
WHO-ACM
Laboratory-confirmed detections
RSV (Country U)
very high
high
medium
low
very low
Week (year)
Seasonal onset
Epidemic threshold

## Supplement S5

### Disease-specific thresholds (AEDSEO) and epidemic thresholds (MEM) for countries A-U, season 2024/25

| Surveillance series | AEDSEO disease-specific threshold | MEM epidemic threshold |
|---|---|---|
| Influenza (Country A) | 42.40 | 356.47 |
| RSV (Country A) | 4.87 | 501.47 |
| ILI (Country A) | 40.46 | 154.28 |
| Influenza (Country B) | 53.51 | 272.76 |
| RSV (Country B) | 10.84 | 110.53 |
| ILI (Country B) | 14.93 | 73.17 |
| Influenza (Country C) | 11.84 | 758.45 |
| RSV (Country C) | 19.28 | 424.06 |
| ARI (Country C) | 62.20 | 242.14 |
| ILI (Country C) | 22.75 | 124.09 |
| Influenza (Country D) | 2.25 | 41.97 |
| RSV (Country D) | 2.20 | 11.39 |
| ARI (Country D) | 75.84 | 427.67 |
| ILI (Country D) | 126.07 | 259.81 |
| Influenza (Country E) | 5.47 | 335.84 |
| RSV (Country E) | 25.86 | 542.54 |
| ARI (Country E) | 556.31 | 2194.27 |
| ILI (Country E) | 122.62 | 398.34 |
| Influenza (Country F) | 12.40 | 30.41 |
| RSV (Country F) | 2.80 | 16.84 |
| ARI (Country F) | 240.64 | 1255.68 |
| Influenza (Country G) | 6.79 | 102.15 |
| RSV (Country G) | 13.26 | 9.58 |
| ILI (Country G) | 1.00 | 23.38 |
| Influenza (Country H) | 8.55 | 224.93 |
| RSV (Country H) | 3.25 | 34.59 |
| ARI (Country H) | 291.98 | 1281.14 |
| ILI (Country H) | 2.66 | 46.90 |
| Influenza (Country I) | 14.40 | 11.25 |
| RSV (Country I) | 1.40 | 5.12 |
| ARI (Country I) | 565.63 | 2031.16 |

| | | |
|---|---|---|
| Influenza (Country J) | 8.74 | 10.65 |
| RSV (Country J) | 1.45 | 13.76 |
| ILI (Country J) | 170.79 | 219.97 |
| Influenza (Country K) | 3.11 | 47.77 |
| RSV (Country K) | 4.96 | 37.32 |
| ILI (Country K) | 1.56 | 19.38 |
| Influenza (Country L) | 19.45 | 59.46 |
| RSV (Country L) | 3.94 | 37.07 |
| ILI (Country L) | 1.88 | 64.77 |
| Influenza (Country M) | 2.23 | 57.75 |
| RSV (Country M) | 2.39 | 12.15 |
| ARI (Country M) | 1240.99 | 2059.95 |
| ILI (Country M) | 18.47 | 76.44 |
| Influenza (Country N) | 3.46 | 129.00 |
| RSV (Country N) | 1.20 | 266.68 |
| Influenza (Country O) | 10.31 | 24.49 |
| RSV (Country O) | 8.22 | 19.11 |
| Influenza (Country P) | 1.97 | 65.19 |
| RSV (Country P) | 1.00 | 2.80 |
| ILI (Country P) | 76.53 | 450.43 |
| Influenza (Country Q) | 37.44 | 231.98 |
| RSV (Country Q) | 5.33 | 120.34 |
| Influenza (Country R) | 2.80 | 10.40 |
| RSV (Country R) | 2.00 | 3.02 |
| Influenza (Country S) | 1.94 | 89.57 |
| RSV (Country S) | 9.23 | 49.11 |
| ARI (Country S) | 971.02 | 2464.71 |
| ILI (Country S) | 2.55 | 29.80 |
| Influenza (Country T) | 13.71 | 339.28 |
| RSV (Country T) | 9.20 | 118.99 |
| Influenza (Country U) | 7.19 | 393.75 |
| RSV (Country U) | 19.83 | 256.87 |

Table S5. Season 2024/25. The table shows 63 surveillance series for ARI, ILI, influenza and RSV. First column is the corresponding disease-specific threshold estimated by AEDSEO and the second column is MEM's epidemic threshold. Influenza and RSV are reported as laboratory-confirmed detections. ARI and ILI are reported as observations per 100,000 population.

## Supplement S6

## Weekly timing of Seasonal onset (AEDSEO) versus epidemic threshold (MEM)

Timing of seasonal onset and the epidemic threshold is estimated by the AEDSEO and MEM methods of season 2024/25 for the external evaluated 21 countries and 63 surveillance series. RSV (Countries D and M) and influenza (Country M) are not included as they did not reach a signal in any of the methods. Influenza (Country D), ARI (Countries D, E and M) and ILI (Country K) are not included as it was only AEDSEO that had a signal in these surveillance series.

| **Surveillance series** | **Seasonal peak (week)** | **AEDSEO seasonal onset (week)** | **MEM epi. threshold (week)** | **Number of weeks between AEDSEO and MEM** | **Number of intervening weeks with significant growth** | **Number of intervening weeks without significant growth** |
|---|---|---|---|---|---|---|
| Influenza (Country A) | 9 | 48 | 51 | 3 | 3 | 0 |
| RSV (Country A) | 6 | 46 | 4 | 10 | 10 | 0 |
| Influenza (Country B) | 5 | 49 | 51 | 2 | 2 | 0 |
| RSV (Country B) | 1 | 45 | 48 | 3 | 3 | 0 |
| ILI (Country B) | 5 | 2 | 4 | 2 | 2 | 0 |
| Influenza (Country C) | 1 | 44 | 50 | 6 | 6 | 0 |
| RSV (Country C) | 51 | 41 | 46 | 5 | 5 | 0 |
| ARI (Country C) | 4 | 36 | 38 | 2 | 2 | 0 |
| ILI (Country C) | 4 | 36 | 38 | 2 | 2 | 0 |
| ILI (Country D) | 5 | 37 | 41 | 4 | 4 | 0 |
| Influenza (Country E) | 4 | 25 | 49 | 24 | 10 | 14 |
| RSV (Country E) | 50 | 26 | 47 | 21 | 11 | 10 |
| ILI (Country E) | 4 | 41 | 1 | 12 | 6 | 6 |
| Influenza (Country F) | 4 | 1 | 2 | 1 | 1 | 0 |
| RSV (Country F) | 8 | 4 | 8 | 4 | 4 | 0 |
| ARI (Country F) | 4 | 33 | 3 | 22 | 8 | 14 |
| Influenza (Country G) | 5 | 51 | 2 | 3 | 3 | 0 |
| ILI (Country G) | 5 | 51 | 2 | 3 | 3 | 0 |
| Influenza (Country H) | 5 | 48 | 1 | 5 | 5 | 0 |

| | | | | | | |
|---|---|---|---|---|---|---|
| RSV (Country H) | 13 | 39 | 6 | 19 | 8 | 11 |
| ARI (Country H) | 5 | 36 | 47 | 11 | 7 | 4 |
| ILI (Country H) | 7 | 37 | 3 | 18 | 6 | 12 |
| ARI (Country I) | 7 | 25 | 4 | 31 | 8 | 23 |
| RSV (Country J) | 9 | 3 | 7 | 4 | 4 | 0 |
| Influenza (Country K) | 52 | 44 | 52 | 8 | 4 | 4 |
| RSV (Country K) | 50 | 46 | 48 | 2 | 2 | 0 |
| Influenza (Country L) | 1 | 47 | 49 | 2 | 2 | 0 |
| RSV (Country L) | 2 | 45 | 49 | 4 | 4 | 0 |
| ILI (Country L) | 1 | 38 | 1 | 15 | 11 | 4 |
| ILI (Country M) | 10 | 44 | 51 | 7 | 4 | 3 |
| Influenza (Country N) | 5 | 36 | 49 | 13 | 5 | 8 |
| RSV (Country N) | 51 | 21 | 51 | 30 | 12 | 18 |
| Influenza (Country P) | 5 | 27 | 49 | 22 | 6 | 16 |
| ILI (Country P) | 4 | 35 | 50 | 15 | 10 | 5 |
| Influenza (Country Q) | 4 | 21 | 48 | 26 | 7 | 19 |
| RSV (Country Q) | 51 | 33 | 47 | 13 | 7 | 6 |
| Influenza (Country R) | 6 | 42 | 47 | 5 | 2 | 3 |
| Influenza (Country S) | 2 | 45 | 52 | 7 | 7 | 0 |
| RSV (Country S) | 7 | 52 | 4 | 4 | 4 | 0 |
| ARI (Country S) | 5 | 28 | 5 | 29 | 9 | 20 |
| ILI (Country S) | 5 | 51 | 2 | 3 | 3 | 0 |
| Influenza (Country T) | 2 | 40 | 52 | 12 | 10 | 2 |
| RSV (Country T) | 50 | 44 | 46 | 2 | 2 | 0 |
| Influenza (Country U) | 9 | 41 | 51 | 10 | 7 | 3 |
| RSV (Country U) | 10 | 49 | 4 | 7 | 7 | 0 |

Table S6-1. The table shows 45 surveillance series with the time (weeks) from when AEDSEO estimated seasonal onset to when MEM crossed its epidemic threshold, and how many of these weeks had significant growth or not.

| Surveillance series | Seasonal peak (week) | MEM epi. threshold (week) | AEDSEO seasonal onset (week) | Number of weeks between MEM and AEDSEO | Number of intervening weeks with significant growth | Number of intervening weeks without significant growth |
|---|---|---|---|---|---|---|
| ILI (Country A) | 7 | 29 | 31 | 2 | 1 | 1 |
| RSV (Country G) | 7 | 48 | 51 | 3 | 3 | 0 |
| Influenza (Country I) | 6 | 3 | 4 | 1 | 1 | 0 |
| RSV (Country I) | 4 | 3 | 4 | 1 | 1 | 0 |
| Influenza (Country J) | 7 | 51 | 2 | 2 | 1 | 1 |
| Influenza (Country O) | 2 | 26 | 27 | 1 | 1 | 0 |
| RSV (Country R) | 12 | 49 | 50 | 1 | 1 | 0 |

Table S6-2. The table shows seven surveillance series with the time (weeks) from when MEM crossed its epidemic threshold to when AEDSEO estimated seasonal onset, and how many of these weeks had significant growth or not.

| Surveillance series | Seasonal peak (week) | MEM epi. threshold (week) | AEDSEO seasonal onset (week) | Number of weeks between AEDSEO and MEM | Number of intervening weeks with significant growth | Number of intervening weeks without significant growth |
|---|---|---|---|---|---|---|
| ILI (Country J) | 8 | 48 | 48 | 0 | 0 | 0 |
| RSV (Country O) | 1 | 47 | 47 | 0 | 0 | 0 |
| RSV (Country P) | 5 | 5 | 5 | 0 | 0 | 0 |

Table S6-3. The table shows three surveillance series with the time (weeks) where AEDSEO estimated seasonal onset and MEM crossed its epidemic threshold at the same week.
